# Association Between Polymorphisms in Gastric Cancer Related Genes and Risk of Gastric Cancer: A Case-Control Study

**DOI:** 10.3389/fmolb.2021.690665

**Published:** 2021-05-17

**Authors:** Yan Pu, Xu Wen, Zhangjun Jia, Yu Xie, Changxing Luan, Youjia Yu, Feng Chen, Peng Chen, Ding Li, Yan Sun, Jian Zhao, Haiqin Lv

**Affiliations:** ^1^ School of Medicine, Southeast University, Nanjing, China; ^2^ Department of General Surgery, Jiangsu Cancer Hospital and Jiangsu Institute of Cancer Research, The Affiliated Cancer Hospital of Nanjing Medical University, Nanjing, China; ^3^ Department of Clinical Laboratory, Jiangsu Cancer Hospital and Jiangsu Institute of Cancer Research, The Affiliated Cancer Hospital of Nanjing Medical University, Nanjing, China; ^4^ Department of Geriatrics, Affiliated Nanjing Drum Tower Hospital of Nanjing University Medical School, Nanjing, China; ^5^ Department of Forensic Medicine, Nanjing Medical University, Nanjing, China; ^6^ Department of Medical Oncology, Jiangsu Cancer Hospital and Jiangsu Institute of Cancer Research, The Affiliated Cancer Hospital of Nanjing Medical University, Nanjing, China; ^7^ Department of Radiation Oncology, Jiangsu Cancer Hospital and Jiangsu Institute of Cancer Research, The Affiliated Cancer Hospital of Nanjing Medical University, Nanjing, China

**Keywords:** EFNA1, gastric cancer, SNP, case-control, biomarker

## Abstract

Gastric cancer has the second highest incidence among all the malignancies in China, just below lung cancer. Gastric cancer is likewise one of the main sources of cancer related passings. Gastric cancer therefore remains a huge threat to human health. The primary reason is absence of high sensitivity and specificity for early detection while the pathogenesis of GC is stayed muddled. During the last few decades, a lot of GC related genes have been identified. To find candidate GC related variant in these GC related genes, we conducted this case-control study. 29 tagSNPs located in 7 GC related genes were included. 228 gastric cancer patients and 299 healthy controls were enrolled. Significant differences were found between the genotype frequencies of *EFNA1* rs4971066 polymorphism between gastric cancer patients and healthy controls. The result indicated that ephrin-A1 tagSNP rs4971066 GT/TT genotypes was significantly associated with reduced susceptibility of gastric cancer development.

## Introduction

Gastric cancer (GC) is one of the most well-known reason for cancer-related demise worldwide with the fifth incidence and third mortality (Jiang and Shen, 2019). The five-year survival rate of serious GC patients is still low. GC therefore remains a huge threat to human health. The primary explanation is absence of high sensitivity and specificity for early discovery. Therefore, to identify potential genetic markers such as polymorphisms in GC-related genes, can contribute the potential early diagnosis of GC. During the present study, we have focused on the following GC related genes: Y-box binding protein 1 (YBX1) encodes an exceptionally conserved protein that has wide nucleic acid binding properties. The encoded protein can bind both DNA and RNA then implicating in many cellular processes. Abnormal expression of YBX1 is related with malignant growth multiplication in various tissue including gastric cancer (Fang et al., 2019). ephrin A1 (EFNA1) is a member of the EFN family. For more than 30 years after researchers find this gene, a ton of proof upheld that EFNA1 assumes a basic part in tumor development (eg., Angiogenesis and progression) (Hao and Li, 2020). Gastrokine 2 (GKN2) is a secretory protein, whose expression level decrease in GC. GKN2 can increase sensitivity of GC cells to the drugs which increase ROS levels in tumors (Zhang et al., 2019). MicroRNA 143 (MIR143) has long to be proved to play a tumor suppressive role in gastric cancer. MIR143 is downregulated in GC cell lines. Ectopic expression of MIR143 resulted in inhibition of GC cell proliferation (Wu et al., 2020). Bromodomain containing 2 (BRD2) plays key role in transcription of genes required for cancer. A new report has shown that BRD2 is a direct target of MIR143–3p and increased expression level of BRD2 in gastric tumors was related with shorter survival times for GC patients (Chen et al., 2019). Leucine rich repeat containing G protein-coupled receptor 5 (LGR5) is involved in tissue development and the maintenance of adult stem cells in gastrointestinal tract. LGR5 can regulates gastric adenocarcinoma cell proliferation and invasion via activating Wnt signaling pathway (Wang et al., 2018). HOXC cluster antisense RNA3 (HOXC-AS3) is a long non-coding RNA that essentially increased in gastric cancer tissues and is corresponded with clinical results of gastric cancer (Zhang et al., 2018).

All of these genes have been reported to be associated with the GC development, and we have selected the tagSNPs in these genes and then conducted a case-control study to investigate whether these tagSNPs could contribute to the GC development.

## Materials and Methods

### Subjects

The study population was composed of 228 gastric cancer patients and 299 healthy control individuals. Patients were consecutively recruited from the Jiangsu Cancer Hospital of Nanjing Medical University between Jan 2018 and Sep 2019. The diagnosis of patients was confirmed by histopathological analysis. Clinical information was obtained from hospital records, including gender, age, smoke, drink, differentiation, location, TNM status. Baseline profiles of the study participants have been summarized in Supplementary Table S1. The controls were selected from healthy volunteers who visited the Sir Run Hospital of Nanjing Medical University for medical examination. Individuals who had a history of diseases were exclude from the control group.

### TagSNP Selection

Population data from 1,000 Genomes phase three were used to screen tagSNPs. A total of 208 individuals from Han Chinese populations were enrolled, including Han Chinese in Beijing, China (CHB) and Southern Han Chinese (CHS). The tagSNP were further screened by using Haploview software. The selected SNPs in the present study were summarized in Supplementary Table S2.

### Genotyping

Genomic DNA was extracted from 200 μl EDTA-anticoagulated peripheral blood using a commercial extraction kit (Tiangen Biotech Corporation, Beijing, China). We performed polymerase chain reaction–ligase detection reaction (PCR-LDR) assay to detect the genotype of the SNP. The primer used were summarized in Supplementary Tables S3, S4. The final production was electrophoresed on ABI3730XL DNA Analyzer (Thermo Fisher Scientific, United States). The SNP was further genotyped by using Genemapper 4.1 (AppliedBiosystems, United States).

### Statistical Analysis

All data were analyzed using SPSS 13 (SPSS Inc., Chicago, IL, United States). Genotype frequencies of the SNP were obtained by directed computing. Genotypic association analysis was performed using SNPstats. Odds ratio (OR) and respective 95% confidence intervals were reported to evaluate the effects of any differences between allele and genotype frequencies. Probability of 0.001 (0.05/29) or less was regarded as statistically significant.

## Results

**FIGURE 1:**
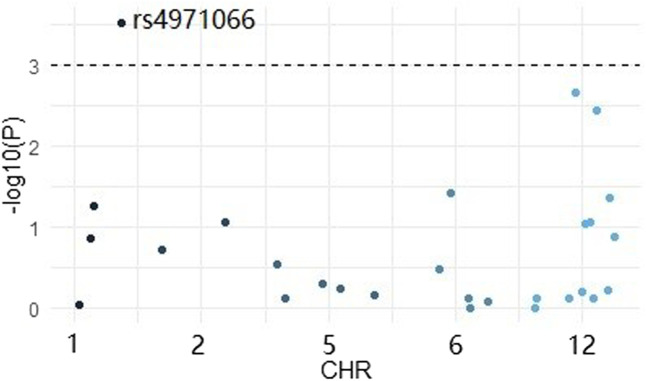
Manhattan plot for the 29 SNPs.

Firstly, we conducted a logistic analysis by using plink software and picked out a significant variant *EFNA1* rs4971066 (*p* value = 0.0003) (Figure 1).

Significant difference of *EFNA1* rs4971066 allele frequencies existed between GC patients (G: 0.89 T:0.11) and controls (G: 0.80 T:0.20). Comparing to G allele, T allele carriers have 0.50-fold reduced risk to develop GC (*p* = 0.0001, 95% CI = 0.35–0.71). Then we performed further analysis based on different genetic models. As shown in Table 1, significant differences were found between the genotype frequencies of rs4971066 polymorphism between gastric cancer patients and healthy controls. Compared with GG genotype carriers, individuals with GT or TT genotype had 0.53 (*p* = 0.002) or 0.23 (*p* = 0.0099) fold decreased risk to develop gastric cancer in a codominant model, respectively. After adjusted by gender and age, GT genotype carriers still had a 0.36-fold decreased risk to develop gastric cancer (*p* = 0.0033). When compared with GG genotype carriers in a dominant model, GT/TT genotypes carriers had a 0.49- (*P* = 3e-04) or 0.34- (*adjusted p* = 0.0013) fold decreased susceptibility to develop gastric cancer. When compared with GG/TT genotypes carriers, GT genotype carriers had a 0.56- (*p* = 0.0044) or 0.38-fold (*adjusted p* = 0.0049) decreased risk to develop gastric cancer in a codominant model.

**TABLE 1:** Association between the rs4971066 and risk of gastric cancer

Genetic model	Genotypes	Patients	Controls	Logistic regression (crude)		Logistic regression (adjusted)^a^	
		*n* = 228 (%)	*n* = 299 (%)	Or (95% CI)	*p* Value^b^	Or (95%CI)	*p* Value^c^
rs4938723							
Codominant	GG	181 (79.4)	195 (65.2)	1.00		1.00	
	GT	44 (19.3)	90 (30.1)	**0.53 (0.35–0.80)**	**0.002**	**0.36 (0.18–0.72)**	**0.0033**
	TT	3 (1.3)	14 (4.7)	**0.23 (0.07–0.82)**	**0.0099**	0.15 (0.02–1.50)	0.094
Dominant	GG	181 (79.4)	195 (65.2)	1.00	**3e-04**	1.00	
	GT/TT	47 (20.6)	104 (34.8)	**0.49 (0.33–0.73)**		**0.34 (0.17–0.67)**	**0.0013**
Recessive	GG/GT	225 (98.7)	285 (95.3)	1.00	**0.022**	1.00	
	TT	3 (1.3)	14 (4.7)	**0.27 (0.08–0.96)**		0.20 (0.02–1.89)	0.14
Overdominant	GG/TT	184 (80.7)	209 (69.9)	1.00	**0.0044**	1.00	
	GT	44 (19.3)	90 (30.1)	**0.56 (0.37–0.84)**		**0.38 (0.19–0.75)**	**0.0049**

a Adjusted for age and gender using the logistic regression model.

b *p* value = 0.0044, multiple testing in a codominant model.

c *p* value = 6e-04, multiple testing in a codominant model.

OR odds ratio; CI confidence interval.

Boldfaced values indicate a significant difference at the 5% level.

Furthermore, we divided the patients by their T status, N status, clinical stages, and multifocality. No significant differences were found between patients with different TNM status (Table 2).

**TABLE 2:** Association between the rs4971066 polymorphism and clinical features of GC patients

Clinical features	Genotype frequency		Or (95 CI)	*p*
	N (%)	N (%)		
T Status	T1 and T2	T3 and T4		
GG	78 (83)	95 (80.5)	1.00	
GT	14 (14.9)	22 (18.6)	1.21 (0.57–2.59)	0.496
TT	2 (2.1)	1 (0.8)	0.25 (0.02–3.27)	0.457
G	156 (89.6)	212 (89.8)	1.00	
T	18 (10.3)	24 (10.1)	0.981 (0.515–1.870)	0.954
N status	N0	N1—N3		
GG	83 (83)	69 (78.4)	1.00	
GT	14 (14)	19 (21.6)	1.62 (0.75–3.47)	0.250
TT	3 (3)	0 (0)	0.00 (0.00-NA)	0.254
G	180 (90)	157 (89.2)	1.00	
T	20 (10)	19 (10.7)	1.08 (0.56–2.11)	0.866
Clinical stages	Ӏ and Ⅱ	Ш and Ⅳ		
GG	107 (82.3)	66 (80.5)	1.00	
GT	20 (15.4)	16 (19.5)	1.25 (0.60–2.61)	0.574
TT	3 (2.3)	0 (0)	0.00 (0.00-NA)	0.293
G	224 (89.6)	148 (90.2)	1.00	
T	26 (10.4)	16 (9.7)	0.93 (0.48–1.79)	0.86
Multifocality	No	Yes		
GG	167 (81.5)	5 (83.3)	1.00	
GT	35 (17.1)	1 (16.7)	0.97 (0.11–8.64)	0.96
TT	3 (1.5)	0 (0)	0.00 (0.00-NA)	0.76
G	369 (90)	11 (91.6)	1.00	
T	41 (10)	1 (8.3)	0.81 (0.10–6.49)	0.84

**FIGURE 2:**
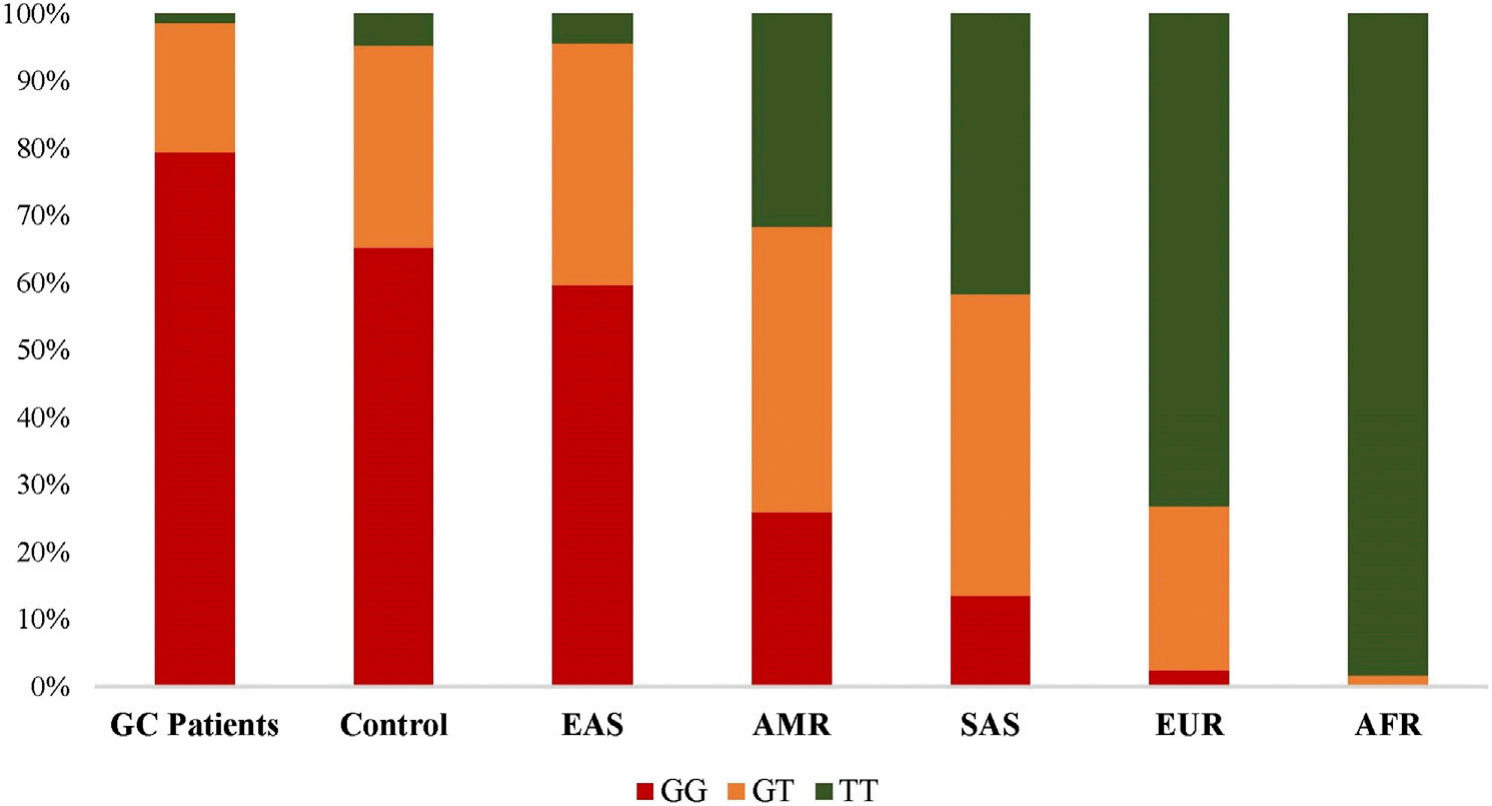
Distributions of *EFNA1* rs4971066 genotypes in studies populations and other ethnic populations.

By enrolling the population data from 1,000 Genome Project, we then compared the frequencies of EFNA1 rs4971066 genotypes in present studied populations and different continental populations. As shown in Figure 2, dramatic differences were observed among populations from different continent. The frequency of GC risk rs4971066-GG genotype is highest in EAS population. Consistently, the highest GC incidence was in Asia and the lowest incidence in Africa (Rawla and Barsouk, 2019).

For the single nucleotide polymorphisms in EFNA1, there are couple papers have demonstrated that rs12904 is a gastric cancer related variant (Li et al., 2014; Lee et al., 2015; Zhu et al., 2015). As shown in Supplementary Figure S1, strong linkage was found between rs12094 and rs4971066 (D’ = 1, r2 = 0.862). This result also indicated that rs4971066 can served as a highly effective genetic marker.

## Discussion

The Eph family have been associated with controlling cell adhesion, migration and spatial organization of multicellular tissues (Anderton et al., 2021). There are at least 16 receptors and nine ligands recognized in various species belonging to the Eph family, which makes this family the largest family of receptor tyrosine kinases (Brantley-Sieders et al., 2006). These receptors can be partitioned into two classes dependent on homology and binding affinities for two distinct classes of ephrins. EphA-class receptors bind glycosylphosphatidylinositol-anchored ephrin-A ligands, which are bound to the cell membrane. EphB class receptors typically bind to class B ephrins, which are anchored to the cell membrane by a transmembrane spanning domain (Shi et al., 2008). Growing evidences have suggested the roles of A-class receptors and ligands in postnatal angiogenesis regulation, embryonic vascular remodeling and tumor angiogenesis (Pasquale, 2005). Expression analysis of mouse xenograft models and human breast cancer or human Kaposi’s sarcoma demonstrated that ephrin-A1 was widely expressed in tumor parenchyma and tumor endothelium (Ogawa et al., 2000). Other studies using inhibitors indicated that A-class receptors are necessary for vascular remodeling in pancreatic islet cell cancer and metastatic mammary adenocancer (Brantley et al., 2002). Scholars found out through a series of experiments in metastatic mammary tumor that membrane-tethered Ephrin-A1 can regulates angiogenic responses from initially distant host endothelium (Brantley-Sieders et al., 2006). The increased expression of ephrin-A1 accelerated the malignant progression of the intestinal adenoma to invasive tumors (Shi et al., 2008). Ephrin-A1 also regulates glutaminolysis through Eph receptor-dependent activation of RhoA GTPases (Youngblood et al., 2016). Ephrin-A1 was recently found it can be targeted by a lncRNA, GMAN, by binding competively to GMAN-AS RNA. Knockdown or knockout of GMAN or EFNA1 in gastric cancer cell lines reduces invasive activity and metastases.

Based on the previous results, it is concluded that the abnormal expression level of ephrin-A1 play a critical role in the tumor occurrence, development and metastasis. As well known, the nucleotide changes in the gene may have additive effect in the function of the specific gene. Not surprisingly, there were several studies have revealed single nucleotide variants in the ephrine-A1 gene that were associated with different diseases. For instances, a SNP rs12904 in the 3′-UTR of ephrin-A1 was found to be associated with gastric cancer susceptibility (Li et al., 2014). A GWAS research of Asian ethnicity has revealed that ephrin-A1 rs4745 and rs12904 were associated with the risk of gastric cancer (Lee et al., 2015). And a SNP rs4745 was found to be significantly associated with type 1 diabetes in a Genome-wide pathway analysis (Lee and Song, 2016). Li et al. identified that rs12904 in ephrin-A1 gene was significantly associated with risk of gastric cancer in a Chinese population. Their data indicated that the OR for carrying AG or GG genotype being 0.65 compared with AA genotype. During the present study, we got a consistent result. Since rs12904 is in strong linkage disequilibrium with rs4971066. The rs12904 AG/GG linked rs4971066 GT/TT genotypes also reduced the risk of gastric cancer. The result of present study demonstrated that rs12904 could be potential genetic marker for predicting the susceptibility of gastric cancer.

The present study has an obvious limitation. As noted in the Supplementary Table S1 that the ages of patients and controls are significantly different. The controls are younger, and they might develop GC in the future. To further validate the results, healthy independent Han Chinese individuals from 1000 G database were also compared. And the results were consistent.

## Data Availability

The original contributions presented in the study are included in the article/Supplementary Material, further inquiries can be directed to the corresponding authors.
