# Supplementary material for: Association Between Polymorphisms in Gastric Cancer Related Genes and Risk of Gastric Cancer: A Case-Control Study
Source: Front Mol Biosci. 2021 May 17;8:690665. doi: 10.3389/fmolb.2021.690665 (PMC8166284; doi:10.3389/fmolb.2021.690665)
Supplement: Supplementary file 1 [file Table1.DOCX]

Supplementary table 1. Demographic and clinical characteristics of gastric cancer patients and control subjects.

|  | Gastric cancer | Controls | |  |
| --- | --- | --- | --- | --- |
| Age (years) | 61.3 ± 9.6 | | 37.50 ± 10.61 | |
| Gender (%) |  | |  | |
| Male | 148 (64.9) | | 209 (69.9) | |
| Female | 80 (35.1) | | 90 (30.1) | |
| Smoke |  | |  | |
| Yes | 52 (22.9) | |  | |
| No | 175 (77.1) | |  | |
| Drink |  | |  | |
| Yes | 27 (11.9) | |  | |
| No | 200 (88.1) | |  | |
| Lauren’s classification |  | |  | |
| Intestinal type | 57 (47.5) | |  | |
| diffuse type | 63 (52.5) | |  | |
| Differentiation |  | |  | |
| Low grade | 78 (36.7) | |  | |
| Middle grade | 130 (61.3) | |  | |
| High grade | 4 (1.8) | |  | |
| Location |  | |  | |
| Proximal | 63 (29.1) | |  | |
| Middle | 106 (49.0) | |  | |
| Distal | 47 (21.7) | |  | |
| Stages |  | |  | |
| Ⅰa | 58 (27.3) | |  | |
| Ⅰb | 16 (7.5) | |  | |
| Ⅱa | 16 (7.5) | |  | |
| Ⅱb | 40 (18.8) | |  | |
| Ⅲa | 57 (26.8) | |  | |
| Ⅲb | 15 (7.0) | |  | |
| Ⅲc | 4 (1.8) | |  | |
| Ⅳ | 6 (2.8) | |  | |
