# Supplementary figures and images for: Association Between Polymorphisms in Gastric Cancer Related Genes and Risk of Gastric Cancer: A Case-Control Study
Source: Front Mol Biosci. 2021 May 17;8:690665. doi: 10.3389/fmolb.2021.690665 (PMC8166284; doi:10.3389/fmolb.2021.690665)

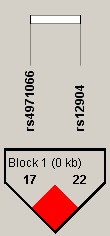

Supplement: Supplementary file 2 [file Image1.JPEG]
