# Supplementary material for: Association Between Polymorphisms in Gastric Cancer Related Genes and Risk of Gastric Cancer: A Case-Control Study
Source: Front Mol Biosci. 2021 May 17;8:690665. doi: 10.3389/fmolb.2021.690665 (PMC8166284; doi:10.3389/fmolb.2021.690665)
Supplement: Supplementary file 5 [file Table2.DOCX]

Supplementary table 2. Detail information of the 29 tagSNPs.

| Chromosome | Position | Gene | ID |
| --- | --- | --- | --- |
| 1 | 42692839 | YBX1 | rs10493113 |
| 1 | 42697875 | YBX1 | rs12044179 |
| 1 | 42686939 | YBX1 | rs3862218 |
| 1 | 155133406 | EFNA1 | rs4971066 |
| 2 | 68949675 | GKN2 | rs4854469 |
| 2 | 68951339 | GKN2 | rs118070474 |
| 5 | 149421424 | MIR143 | rs117675324 |
| 5 | 149414478 | MIR143 | rs17796690 |
| 5 | 149419838 | MIR143 | rs34060527 |
| 5 | 149426373 | MIR143 | rs4705341 |
| 5 | 149412643 | MIR143 | rs78278727 |
| 6 | 32973918 | BRD2 | rs115417321 |
| 6 | 32972365 | BRD2 | rs11555940 |
| 6 | 32975331 | BRD2 | rs150850682 |
| 6 | 32969659 | BRD2 | rs72865868 |
| 6 | 32972777 | BRD2 | rs78280497 |
| 12 | 71553626 | LGR5 | rs11178846 |
| 12 | 71553393 | LGR5 | rs1148985 |
| 12 | 71563353 | LGR5 | rs1280606 |
| 12 | 71542831 | LGR5 | rs1298467 |
| 12 | 71482785 | LGR5 | rs147361802 |
| 12 | 71478682 | LGR5 | rs17814919 |
| 12 | 71453947 | LGR5 | rs4132612 |
| 12 | 71504147 | LGR5 | rs4760939 |
| 12 | 71450132 | LGR5 | rs530605210 |
| 12 | 71512836 | LGR5 | rs533309272 |
| 12 | 71527314 | LGR5 | rs66814253 |
| 12 | 71570688 | LGR5 | rs7976133 |
| 12 | 53982480 | HOXC-AS3 | rs12422555 |
