# Supplementary material for: Association Between Polymorphisms in Gastric Cancer Related Genes and Risk of Gastric Cancer: A Case-Control Study
Source: Front Mol Biosci. 2021 May 17;8:690665. doi: 10.3389/fmolb.2021.690665 (PMC8166284; doi:10.3389/fmolb.2021.690665)
Supplement: Supplementary file 6 [file Table4.DOCX]

>rs10493113
AGATGGACATTAGGTACAGCCATCATTTCCCTAGGCAATCCTAATCATCTAGCTTGAAAGGTCAGTTTTCAGTCACTTGCCTTAAGTCGCTTTTACAGTTTACCTGTTCTGTTTTTTTAATTATCCTGTTCCCATCAATTAAAAATGTTCTTATAATAATCCAAGCTATAATTTACAGGTCAGATTTAAAGTTAATTTTATGTGGATCCTTTGATGCATATGTTGCCCCCAGTTCAGCAAGAAGCTAACAGTCAGTAAGCCAGTTTGTCCAGTTCATTGTAACCTTTGTTCCCTTTGTCATTAAACGCTAGAGCTACTACTGAGCTTTTTCTCACTCTGACCTGACGGAGTAAAGAGGGTGAGGCAGGCCACTGCAGGCCTGGAAATCCGTTTCCTTCTCCAGTGGTGATTCTTGCTCCTCGTGTTATCACCTTAATCCCAATATCcaggctaaaaagtgagattattctctaccccctcgcttctgacatccagtcatc[Y]acaaagtttctcattttgcctcaaaaagagatctcaaatgtctttttatacatggctccgtggtctaaaccttcatcagtccttgtctggacttgtggagtagcttttataactgggctTTTcttacattcaaacataccttatcacccaaaggtcacagttcccattaggtttcactctttgcgttatacattctgttggtGGGGAGGGGGGTTCTCATTTTTATGATCTCAAGGAAAGTTGGTAGTTGGGAGATGCAGGATAAGGTGAGAATGTAACTACTACTTCTGTTGGTAACCATGCTCTAGAGGTAAGAGGAGGGTGTGCTAGAACAGTTAATGTTTAATAGGATAAGGAGGGGCAGAATATATTTTGCTAGTCATTGCTACCCTCTTAATTATGGCTCAGTAGGTCACACTTTAATTTCTTAGACTTTTTTTTTTTTTTGCCTATCTTACGGGTAATGAGGCTACAACTGTTTGCAACTGCTGAAGTCTTGA

OLIGO_Name Start Length Tm Gc com 3'com Rep seq

rs10493113F 352 21 66.78 61.9 4 2 0 AAGAGGGTGAGGCAGGCCACT

rs10493113R 644 22 65.58 54.55 3 2 0 GGGAACTGTGACCTTTGGGTGA

ProductSize:314,PAIR ANY COMPL: 4, PAIR 3' COMPL: 1,Score 1.33 Rank Left:1 Right:1

TITLE:rs10493113 NUM:1 SNP:0 Strand:1

rs10493113RC:TTCCGCGTTCGGACTGATAT TCTCTTTTTGAGGCAAAATGAGAAACTTTCTG 68.68

rs10493113RT:TACGGTTATTCGGGCTCCTGT TCTCTTTTTGAGGCAAAATGAGAAACTTTCTA 66.80

rs10493113RP: GATGACTGGATGTCAGAAGYGAG TTTTTTTTTTTTT 63.49

>rs11178846
TGCATTCCCTGAGGCACCTGTGGCTGGATGACAATGCGTTAACAGAAATCCCCGTCCAGGCTTTTAGAAGTTTATCGGCATTGCAAGCCATGACCTTGGCCCTGAACAAAATACACCACATACCAGACTATGCCTTTGGAAACCTCTCCAGCTTGGTAGTTCTGTAAGTTTTATTGATTTTGCTCTCTTTTAACAGTTTCTAATGTCACTGGAAGACCTTGGCCTTAAAATGCTGGCTCTTCAAAGCTCAAATGGGGACATTTTAAGGGAGGAAAACCTCCTGAGCCCCTCCCCAAAATGACTTATAAATGCTCCAATTGTGTAAATGACttaaacaatacttatcaattacctgctagatgccaagcacagcagggcatacaaagataatgaaatatgacctgtgccttagagaagcccCTTGCATGTTGCACAAGGAGTCACCGCACTGTACAGTTAGTACAGTAACCTAAGTTAAACATTCCTTATTTTAAAAAATA[D]TTGATAATTCAGGTCAAATTGAAAGATGCTCTTCTTATTATCTAATAAGAACTTTGTGAACCCTTACCAAGGAAAGAGAAGTGAGAACAAATGAtgtaactgctcaatgagttctttttgcccactgcccaaatagagccaatttattaagacaagagaattgcaataaagagtaacacacaaggccggcacagtggctcatacctgtaatcccagcactttgcgaagccaaggcgggtgtatcacttgaggtcaggagttcgaggccagcctggtcaacatggtgaaaccccacctctactaaaagtacaaaaattagctgggcgtagtggcacatgcttgtaatcccagctactagggaggctgaggccggaggatcaattgaacccaggttgcagtgagccaagattgtgccactgcactccaccctggggaagagagtgagactctgtctcaaaaaaaaagaaaaaatagtttagcacacatacagccagctcaat

OLIGO_Name Start Length Tm Gc com 3'com Rep seq

rs11178846F 430 23 66.86 56.5 3 2 0 GCACAAGGAGTCACCGCACTGTA

rs11178846R 609 22 66.26 54.5 3 2 0 GGGCAGTGGGCAAAAAGAACTC

ProductSize:201,PAIR ANY COMPL: 6, PAIR 3' COMPL: 2,Score 0.65 Rank Left:1 Right:1

TITLE:rs11178846 NUM:1 SNP:0 Strand:2

rs11178846RT:TCTCTCGGGTCAATTCGTCCTT GAGCATCTTTCAATTTGACCTGAATTATCCAA 67.51

rs11178846RA:TGTTCGTGGGCCGGATTAGT GAGCATCTTTCAATTTGACCTGAATTATCCAT 66.80

rs11178846RP:TATTTTTTAAAATAAGGAATGTTTAACTTAGGTTACTGTAC TTTTTTTTTTTTTTTT 63.17

>rs1148985
TACACATGAATGAGAAGGAGAGATCATCTGGAGAGGAAAATGCTCCAGATGTCAGGGCTCTCGGGGACCCCAGGCCCTGTTCCACTCTTGTTCATCATGCATGGGGAGGGTTTTCCTGCAAAGTTGACCTTCTGCTTGGGCTTTGCTCTCTTTTCCATTCTTGCTTTCTTTCTTCCACAGGCGTCTGGATGCTAACCACATCAGCTATGTGCCCCCAAGCTGTTTCAGTGGCCTGCATTCCCTGAGGCACCTGTGGCTGGATGACAATGCGTTAACAGAAATCCCCGTCCAGGCTTTTAGAAGTTTATCGGCATTGCAAGCCATGACCTTGGCCCTGAACAAAATACACCACATACCAGACTATGCCTTTGGAAACCTCTCCAGCTTGGTAGTTCTGTAAGTTTTATTGATTTTGCTCTCTTTTAACAGTTTCTAATGTCACTGGAAGACCTTGGCCTTAAAATGCTGGCTCTTCAAAGCTCAAATGGGGACATTTTAAG[R]GAGGAAAACCTCCTGAGCCCCTCCCCAAAATGACTTATAAATGCTCCAATTGTGTAAATGACttaaacaatacttatcaattacctgctagatgccaagcacagcagggcatacaaagataatgaaatatgacctgtgccttagagaagcccCTTGCATGTTGCACAAGGAGTCACCGCACTGTACAGTTAGTACAGTAACCTAAGTTAAACATTCCTTATTTTAAAAAATAATTGATAATTCAGGTCAAATTGAAAGATGCTCTTCTTATTATCTAATAAGAACTTTGTGAACCCTTACCAAGGAAAGAGAAGTGAGAACAAATGAtgtaactgctcaatgagttctttttgcccactgcccaaatagagccaatttattaagacaagagaattgcaataaagagtaacacacaaggccggcacagtggctcatacctgtaatcccagcactttgcgaagccaaggcgggtgtatcacttgaggtcaggagttcgaggc

OLIGO_Name Start Length Tm Gc com 3'com Rep seq

rs1148985F 452 23 65.72 47.83 4 0 0 TGGCCTTAAAATGCTGGCTCTTC

rs1148985R 609 22 66.57 59.09 4 3 0 CCTGCTGTGCTTGGCATCTAGC

ProductSize:157,PAIR ANY COMPL: 4, PAIR 3' COMPL: 0,Score 1.85 Rank Left:1 Right:1

TITLE:rs1148985 NUM:1 SNP:0 Strand:2

rs1148985RG:TCTCTCGGGTCAATTCGTCCTT GGGGCTCAGGAGGTTTTCCACC 68.39

rs1148985RA:TGTTCGTGGGCCGGATTAGT GGGGCTCAGGAGGTTTTCCACT 66.17

rs1148985RP:CTTAAAATGTCCCCATTTGAGCTTTG TTTTTTTTTTTTTT 64.82

>rs115417321
TCATTAAATCTTTTGCTTTCTTTGGGTGGGTGGGTTGTGTGTGGTGTTTGTTGGTGCAGGGATTGTTTTTTCCTAACATTAAAAGTTTGATTCAGGGCAGGAGGGTAGAGCTAAGGTTCCTAGTTCAGCTCTGCGATGTAAACAATGAGATTCCCATATGATGTTTTAATTCTTAGGTGGTAGGAAAGACTGATCGGAGGAGCACCAGAGGGACTGTAAATGAACCACTGTTAGCGTTTGGTGTCCGGAGTTGGTGCTACAGGGGGAACTGGTAGTGGAATCGTGTTGTGTAGTGGGTGGGTGGAAGGGGGCTATCACTTGGTGACCTTGACTGTTTTGTACGGCTTTTTGACTTCCTTGGAGTGAGGAGACTCTGATTTGGTGCGAATAATTTTGAGGGCCTGGAAGTTACGGGCTGTGAAGTCTGACAAATTCTTCCTTGTCTGAATTTGTTTTTAAGTTGATATGGTTCTTCCTCTGGGTTTCTAGTCTATGTTCTG[N]TGTGGCGTGAACTACCCAGACCTTGTGGAAGATGGTGCTCTCTCTTCTATCTAGGTGGATTATTCTGTGTCTTATCAGCATTTTATGGAATTTTTTATAGCCATAATTTGTTCTTTTCCTCCTTACCGGCGCTCAACCACCATGGCAACCACCAAACCCCTAGTGAGGAGGAAGCTTGGGGTTTGAGTTTCTTAACTCCACCCATTTTGCTTAAGCCCCATCCCCATAGGGCTGTAGTTCTGAGATGTCGTGCCTTGTCAGAAACAATTTGGGAGTTTTTTAAAATATGAAAAAGAACAGATAGAGCCTATCAGACTTAAGAAGGTGGGATCTAGATAGTATACTAAAAATATTAATAAAAGGAAGGCGGGGCCAGCAATAAAAGCTCCACAGATTGTTTGGATATTGTTTCTGCTTAAGAAGCACTTGGCATAAGCTTAACCACCTCACTAGGGCCAGCACCTGGATTCATCAGACTATTGTGCAGATGCACTTTTTCC

OLIGO_Name Start Length Tm Gc com 3'com Rep seq

rs115417321F 384 22 64.32 45.5 5 3 0 CGAATAATTTTGAGGGCCTGGA

rs115417321R 665 22 65.95 54.55 6 0 0 TCAAACCCCAAGCTTCCTCCTC

ProductSize:303,PAIR ANY COMPL: 5, PAIR 3' COMPL: 1,Score 1.49 Rank Left:1 Right:1

TITLE:rs115417321 NUM:1 SNP:0 Strand:2

rs115417321RC:TCTCTCGGGTCAATTCGTCCTT GGTCTGGGTAGTTCACGCCAGAG 67.24

rs115417321RT:TGTTCGTGGGCCGGATTAGT GGTCTGGGTAGTTCACGCCAGAA 67.54

rs115417321RP:CAGAACATAGACTAGAAACCCAGAGGAA TTTTTTTTTTTTTTTTT 63.28

>rs11555940
CCACCCTCTTTCCCTGGAGTCGGCGGACCACAGCTCAGCCAATTGGCTTGGAGATGTGGCGGGTTGCCACTTCCCTGTGGGTCTCTGCGGCACTCTTCTGCCTGGTGACTGACACCTTGGAAATGAAGTTTATGACGTCATCGTTGCGGCTGGCCAATAGAAAAAGCTCCCGCGGAGAGGTGTTCCTTCCCCTTCGACTCAGCTTCTTCACCCGCGTGAGCGAGCGCGCGCGCGCGGAGGGGGTGGGGAAAAGCTCAAGCAGGGTGGCGCGCATGAGCGGCGAAGCTCCTCCTCCCCGCCTATATATAAAGGGCTGGCGCGGGGCTCGGCGGCGCCATTTCGTGCTGGAGTGGAGCAGCCTCTAGAACGAGCTGGAGGATTCTGCCTACCGATACAGAGCCTTCGAGTCGTCCGGGGCCGCCATTACAATCCACCTCCATCCGCTTGGAAATGGCCTTCGTCCCGGCCTATGACTGGTCCCAGCGGGCAGTACAGACCCC[B]TAGAAGCCCCTGGAGCTCCCCTTTTTCGGGCCCCGCCCAATCCTCGGAGTCTGTCCACCCCCTCTACTCCGCCCTCAAGAGGATTTCAAAGATGGAGGCGGCGGCTCCCTAAACCACTTTTCGTGTTCATCCGCCTCCATCCGAGATCGAAACGGGACCTCGTCGGCCCCGTAGGGGCCCGACAAGAAGAGGGAATCCCTGCAGACCAACAGCGGGCTATATTGACGACGGTGTCTGAGATCGGGGACCGTCTTTTGAAGAGTCAGTCCCTCCTTAGTTGCCCGCCTCAGCTGAGGCCGCCGCCATTTTCTTGCTGTCCGCCGTCTGCAGAGCGCGCCAAGCTGCCCGGAGCTCTCCGAGAGGCCCCAAAGAGACTGCTTTCGTGCCGGCCAGGCAGGGGGTTTGTCGCCTGGAGGCCCAAGAGGAACGGCCTCCCCCCAACTTAGCGGGTTATGCTGGACCGGGCGGTGAGGGGAACCGAGGCCACCCGGACTTTCCGC

OLIGO_Name Start Length Tm Gc com 3'com Rep seq

rs11555940F 388 23 66.07 56.52 5 3 0 CCGATACAGAGCCTTCGAGTCGT

rs11555940R 583 22 65.73 63.64 6 2 0 CCTCTTGAGGGCGGAGTAGAGG

ProductSize:195,PAIR ANY COMPL: 5, PAIR 3' COMPL: 2,Score 1.34 Rank Left:1 Right:1

TITLE:rs11555940 NUM:1 SNP:0 Strand:1

rs11555940FC:TCTCTCGGGTCAATTCGTCCTT CAGCGGGCAGTACAGACCACC 70.31

rs11555940FT:TGTTCGTGGGCCGGATTAGT CAGCGGGCAGTACAGACCACT 67.98

rs11555940FP:TAGAAGCCCCTGGAGCTCCC TTTTTT 65.09

>rs117675324
agaattattcctgcctctggcgacatcaGCGAATGTGCCTTGAGTTACGGGAGTGTTTTGAAGGCTGAAAGCTTTGTGTACACGGAAGGGGTCAAAGACAGCCACAAGCCTGTCCCTTTGGTCTTGGTCATCATGTTAACCCCAGGTTTCGATTTAATCCTACATGCTCCTTTCTTATGTGTGAGGCTGAGGTGGGGGCATTGGTCAGAGGAGCCTCTGGGAAATTTCCTTTCAGGACAGTTCTTTTGTTAAGGGGCCTGGGTGTGCTGAGAAGGGAAGGTGGCTCCTCCTGCTCTTGGAGCTTGGAGTTTCTCACAAGCTCTCCCTTAAAAGCCAGGCAGGCCAAGAAGGGATAATGGCTGCTCAGGGCCCAAGGGGCAGGATCTCTGAGGCCCGGAAGATCTCAGTAGGTTTAGCCCAAGAGTATCCAGAAGTGGCCCAGGCCCATTCTCCATCCCTCAGAGCCTGGCAGCCTGGTCAGGACCCTCAAAGGCCATGAC[Y]GTACCCGTAGGGCACCCTGGTGTCCTTCTGACAGGTGGGATCTTTGTTTCTTACAGGCAAGGGCGCTAGCTGGTGGGAGCCACCCCGCCATGCTGATGTCAGAGAAGCAAGAACTCTGGAGAAGCAGCCTCCTGGGACCAGAGGAGGGCCAGCAGCAGGCAGCCCGGAGACAGAACTGTGAGCCCACCCCACCTCCACCCTTCCGGCACCCACATCCGCTCCCCACCCCAGCCCACCTGCAGGTGGCTCCTGGAGTATCCTGGGTCCCTCAGGATAAACTCCCCACCCTAACCTCAGAATGACCAAGAAACTGGGCCTGAGGATGACAAGAAGCAGAATGGGCCTTGGTAAAGGAAGACCCCCCACTGATTGGTGGCAGTGCGGGCAGTTCAGGGCTGTCAGCGTCTGGGTTGTAGGTAAAAAGAAATGGAACCCAAGAAGGGAAATCCAACTAGGAGCCAAGAATCCGGTCTACCCCATCCTCCTTACGAAATAGCAAT

OLIGO_Name Start Length Tm Gc com 3'com Rep seq

rs117675324F 336 22 65.98 54.55 4 1 0 GGCAGGCCAAGAAGGGATAATG

rs117675324R 541 22 65.89 59.09 5 1 0 TCCCACCTGTCAGAAGGACACC

ProductSize:205,PAIR ANY COMPL: 4, PAIR 3' COMPL: 1,Score 0.13 Rank Left:1 Right:1

TITLE:rs117675324 NUM:1 SNP:0 Strand:2

rs117675324RC:TTCCGCGTTCGGACTGATAT CCAGGGTGCCCTACGGGTTCG 69.76

rs117675324RT:TACGGTTATTCGGGCTCCTGT CCAGGGTGCCCTACGGGTCCA 68.34

rs117675324RP:GTCATGGCCTTTGAGGGTCC TTTTTTTTT 64.04

>rs118070474
CAGATATCTCAGAAAATGTACACCTAGAGACACTGAGTGGCCACAGATGGTCAGCACTCCCTTTCCACGCACACATTGATTGGCTCACACATTGGCtcatttaacaggcatttgttgagttcattctatttgccagacacagggtgctacaaTTGCCACCTAATTCCCAACTCTTTCTAGCATTATAACCATGTAGATAGTAGGAGAAACCCGGGGCAGATGAGCAGTGAAGGATTCATTTCACTATGGAAATACTCTGGTATGATTCACACTAGCTTTGGATAACAGTGGTCAAATTGTGTCCTCTAAAAAAAtaattaattaattaattaattTTAAAAAACTTTAATCCCTTAAGACTGAGAGGGTAATACAGATTTAAAATGAAtcatgggtgataaagacctgacaggttcctaagggaaactatgaaaatagaatgcttgggagcacaattaacttttgaggctgacatcaagtaaaacCCCCATATTCTCCTA[B]GCCTTAGTGATACTATCACACAAGTGCTGGGCCCACAGGTCGTGAGCTGACATTGAAGCAGTTCTGATATTTTGCTGTAGTTCTGTAAGAGTCTGTTCCAACATGCTAACCCAGGTTTTGTACCCTCTCTTCTGAACATCAGTGAACTGTACTATCGCTGAAAACTACATCAGTACATAGTTGACTGCACTGTCATTTGAAAACTGCTGATGAAAGGAGTCTTGGGGAAGGGGGATATTTTTAAGAACTGTCAGTGGGACTTTCCTACTTCCCTTTCCATCCCACCCTTAATATTTCTCTTCTGGGGACTGGGAGTAGAGCATCTAACAAAGACCTGTGTGCTGATATTCAGCCTTCCATAGGGATACATCTTCTCTTACCACAGACCAGCTCCAGCAACATATGATACTCCCGTATATTCTAGTTAATGAGTCTAAAATAGTGGTTTGAACAAGTAGTCGAAGGACTAAACCACTTTAATGGTATGATTAGAGAGAGCT

OLIGO_Name Start Length Tm Gc com 3'com Rep seq

rs118070474F 199 22 66.67 59.09 6 2 0 GTAGGAGAAACCCGGGGCAGAT

rs118070474R 535 22 66.14 59.09 6 2 0 GGGCCCAGCACTTGTGTGATAG

ProductSize:336,PAIR ANY COMPL: 4, PAIR 3' COMPL: 0,Score 0.81 Rank Left:1 Right:1

TITLE:rs118070474 NUM:1 SNP:0 Strand:2

rs118070474RG:TTCCGCGTTCGGACTGATAT CAGCACTTGTGTGATAGTATCACTAAGTCC 66.79

rs118070474RT:TACGGTTATTCGGGCTCCTGT CAGCACTTGTGTGATAGTATCACTAAGTCA 66.37

rs118070474RP:TAGGAGAATATGGGGGTTTTACTTGATG TTTTTTTTTTTTTTTTTTTTTT 64.28

>rs12044179
TTTCATCAGATGCCTAAGAGGTGAACCTATTAAAAACAGCTTGTATAGATGAGGTCTGCTTTATACATAAATTTTTTTAGAAAATGATGAGGGTTTGGAATTTCAAATTTTCTGTCCTTGAACAGGTAAGATAATATTTTAAACCACACCTTTTAAACCTGCCTCCTTGGTAGGGAGAAAGGAAAGCATTAGGAGTATCTGAATTGTACCATGGCATTAGTGAAGAATCAACAGTGTTATCACTTGAAATGGCATACATTATCTTCCACTAAACACTTCTCAGTTTAGATATGTACCCCCTTTTTTTTGGAGTTGGGAGTTGTCAGCTCAAAGTTGCAAAATGAAATTGCTCCACCAAGAATTTACATTCTGCGGGGGGAAATCCAGTCTGTCGTTAGACAACTATCTACTTGAGTGGGTATTAGAAATCTTATTAATAGATGTCATTATTAGAGTAATCGAGGAAAAATTCCATGAACCAAGCATATTTTAGTCCATGC[Y]GTAGTATACTTTGAGGCAGTGTTCCCAGTCCCACGGTGGTAGGATAGGATGGTGTAATAGTTGTTTCTCTAAGGGTTTGATATCAAAGCATTGAGTTTTCATAGGTGATCCCCAAAAGCTGACAGGTACCTCTGAAGAATCAAACCAATACAGTAATAACGCATCTTAACTGATCACTTAAATCGCAACTTTTAAATCCCCAAATTTTTAAGTGTCAAGTTTATTATTCTAGAAGTATCCAACATATCCAAAATATACTGTGAGTCTATAAGAGGGGGTTTCTGTATATAGGTTTTTAAATTTTACCAAAGTTGGAGCACTTAGTGAAGTTTTAGGAACTAGGGAATCTAGTCGAGGTTGTATTACATGTATTTATCAGCACATGTACATTGAGAGTTTTGCAAGAGCAGCTGATGTCATCATATTTTTTGTATTAATATGTCTTGTCCTGAGTTTTCAGTTAAAAGAGCTGAGGTCCTAACTGATCACTACCACAATTG

OLIGO_Name Start Length Tm Gc com 3'com Rep seq

rs12044179F 361 22 65.60 45.45 3 2 0 TTTACATTCTGCGGGGGGAAAT

rs12044179R 532 22 65.81 54.55 3 0 0 GGACTGGGAACACTGCCTCAAA

ProductSize:171,PAIR ANY COMPL: 3, PAIR 3' COMPL: 1,Score 0.59 Rank Left:1 Right:1

TITLE:rs12044179 NUM:1 SNP:0 Strand:1

rs12044179FC:TCTCTCGGGTCAATTCGTCCTT CATGAACCAAGCATATTTTAGTCCATTCC 68.60

rs12044179FT:TGTTCGTGGGCCGGATTAGT CATGAACCAAGCATATTTTAGTCCATCCT 66.79

rs12044179FP:GTAGTATACTTTGAGGCAGTGTTCCCAG TTTTTTTTTTTTTTTTTTTT 63.50

> rs12422555
TGCAGCCCCTGGATTTTCATGTTCCCAAGTTGAGGTAAGAGGGGGGAGCCCCAGACGTAGGCATAGGCATAGGTGTCTCAGCGCCCTCGGAAAGTTGGGGAAATGGATTAATAAAAGTCCATATGTGGGTGATTCTAGCAACACAGGCCTCCCCTGGATCCTAATCCAGTTGCCAAGGGCACAGGCTGAAGGATCTTGGCCCTGTGGGGCAGGGCGGTGGGTGCCTGGCAGTGGCGGCAGGCGGGGTCGCTGTGGCGGCCCGCATGCCTTTGAACGCCAGGCCCGGACAATAGGACCGAAACAGCCTTTGCTCGTGGCCTCGGGAAAGACCGAGCCCAAGGGGGCAGGGCTGGGACCCAGGCCCGCACAAATGCTCCCTTCGCCAGGATAAAACACTCACTTTTTCCTTCCTTTTACCTCCACTGAAGCAGAATCTCAAAGTCTGTGAAAACCCAACAAGTTATTCAGGTCTGCGTCCGAGCGGGCTCTAGTTTAGCAGC[H]CCGGGTTTTCGTTTATCTTGCGTAATTAGAGGCAAACATTTGTCCCACCGGGGGTCCCACTTAGGCTGATGAATTTGATTTCTTTTTTCATTAATCGGCCGTGTTTATCATACTTCTAATTATTTCAACTTTAAACACTATTCTGTCTTGTGAGGAGGTGGGAGTCGAGGCGGGAGGCTCCTCCCTGGAGTATTATTTTCATTAATGATGGCGATAATTCCGAAGCGACTCTGTCCTCTCCCTTGGCTAAGGTGAGAGGTGGGCTTGGAAAGAGGTTGGGTGGTGGGACCCCTGAGGCCGATGGGTCTGCTCCCTGTCTGCTGGTTCCCCAGGCTACGCCTTGGGGCTTGTCTGAAGTTTATCTCCTTCACCCTTTCCTGCCGACTCCTTCACCCCCTCCCAGGCTGGGCTCAAACCCTTTGCGGCTCTTCCCTGGAAACCTTTCTCTGATCCCAGCCTGTCCTGGCTGGCCTGGGCAGAGGCTCGGGTAGCACTGTCCT

OLIGO_Name Start Length Tm Gc com 3'com Rep seq

rs12422555F 368 22 66.08 50.00 3 2 0 AAATGCTCCCTTCGCCAGGATA

rs12422555R 604 24 65.10 37.50 6 0 0 CACGGCCGATTAATGAAAAAAGAA

ProductSize:236,PAIR ANY COMPL: 3, PAIR 3' COMPL: 0,Score 2.98 Rank Left:1 Right:1

TITLE:rs12422555 NUM:1 SNP:0 Strand:2

rs12422555RT:TCTCTCGGGTCAATTCGTCCTT CGCAAGATAAACGAAAACCCTGA 67.96

rs12422555RA:TGTTCGTGGGCCGGATTAGT CGCAAGATAAACGAAAACCCTGT 67.06

rs12422555RP:GCTGCTAAACTAGAGCCCGCTC TTTTTTTT 63.89

>rs1280606
TGTGGTATTTGTCACAAATGAGCAGGAGTCCCAATTAGCCTTGACAACCAGAAGAAAAAAACTATTCATTGTTTCTGAAATGTTCTGGCAATATTTTGGCAAACTAAGAGTTTTAAAGTACATTGACAATAAACATTAGCCCTCATTAGAGCGTATTTGCGTATTAAGAGGTTGGCAAATTACATTGCCTTTGCTCCTCCTTCTGCAGCCACAGTACTGGTTTTTCATCACTCTTTTGATATGCTAATACTCCCTGAGGAACTAGCGGCGCCAACCCCAACTCTATATATATTAGACCTCCTATCCACTATTTGACAGTGGTGACCACTTTCTCCCATAAACTCTCCTCTCCTTGCTCCTGCATCTGTTTCCATCTCTTCAAAGCCCGCATTTCTGTGAATGCTTTCCAGGTCCAGTTGGCTACCCACTCCTCATTTCCACCTCTGTAGTCCCCATCCTATCCATACCTCCTGACCTCCACTGCACCAGCCTCTCTGCCA[R]CCATGACATCCAGATTTTCACCAGTCTGCGTGAGGCTGGCCTCCCTCCCTTCACCGTTGGCAGCACTCATCTTCCTTGAAGCACGGCAACCATCTTGTTACTTCTGCTGTGAAATGTGCGTTCAGGATGTCTATTTACACTAAATGCAAACTCCTCATCCTGACATTCACAGTCTAAGGCCACACATGGCCTAAGCCAACCCTGTCAACCAGTTCCCCAAAGCTCTCCTCATTCACCCTAATTCATAAACCAGACTGACTAAGTTATGTTCACGGAACAAAGCTCTTTTCTTACTTCCTTTGCTCCTACGATCCCATTCCCTCACTATGCTTCTTGAAATTCTTTCAGGGCCCATTTTAAAAGCCATTTCTTGAAGTTTTTTATAATCTCTCCCGAACATATGAGAGAGTTCCCTCCTGAGAACCCTAAAACTCCTTATGTGTGTTTATGATACTTCTGCCTTATGCACACAAGCATGTATATTCATACCTGCCCAgtgt

OLIGO_Name Start Length Tm Gc com 3'com Rep seq

rs1280606F 409 22 66.26 63.64 6 2 0 GGTCCAGTTGGCTACCCACTCC

rs1280606R 628 22 65.70 50.00 4 2 0 TCCTGAACGCACATTTCACAGC

ProductSize:219,PAIR ANY COMPL: 4, PAIR 3' COMPL: 0,Score 0.56 Rank Left:1 Right:1

TITLE:rs1280606 NUM:1 SNP:0 Strand:1

rs1280606FG:TTCCGCGTTCGGACTGATAT TGCACCAGCCTCTCTGCAAG 67.45

rs1280606FA:TACGGTTATTCGGGCTCCTGT TGCACCAGCCTCTCTGCGAA 67.79

rs1280606FP: CCATGACA[W](http://www.ncbi.nlm.nih.gov/SNP/snp_ref.cgi?rs=rs1280607)CCAGATTTTCACCA TTTTTTTTTT 64.13

>rs1298467
gtgcagctgaagcagaagtgaggtggagagacgtgcaagaagagcacatctagtgaggcaggtggaggccagaccccttgaaagatatttgggacatgtcaaggatttcagtctttactgtaaaagcaatgggaatctgttggagggttttaaggctggcaggtgggggtactcagatctgtgtcattgcaatagtctaggagagaaaggagaacttcatcaggttagggacggtggagatgaagaaagggagatggattcgagaaatatctaggagatgaaattaccagtgtttggtaatgaattggctatgaagaatgaagcagaaagaagtgtggagaataatttctaagtttctgatttatacagccatgtgcttactgaagccagtcactgggcaagacacactgggagacggctatacatatttggggagtgtgtggttccaggataatgagtttggtcttgagcacttgtgtttgaggaggaacaactaagta[S]agatgtcacatagtttgaaatccagatctggaTGGAAAGAAAAAAAAAAAGTAAAATCTAGTTTAGGTTCCCCAACCATGCATTCCTCTTCAcctaacacttcattataatgattgatttgtctgactaccctttagaaaacatgctccataatggctgggaccatgcctccctggtttactgctaaatcttcagcaccagtagagtagctaggacaaagaaagcactAGGACATACGTATCAGACTGAACTGAAACCTCAAACTCTTAAGTGTTGATCTAGACAGCTGCtcagacaagatcgggcacattcaaggtgatagggccataggcTAGACAGCCACTCAAGATCAGTTCATCTCCCCTAGTTTAACTGTAACCTGCATTCATTCATCTCCCTGGCTCCAAACTCTTACCTCTGTACCCTGGCTTTCCCACATGCCTTTCCTGGGCTGGGAGTTGGACCCAGTGGTTCCTGCAGAGATTTTAGAGTTGATCTAT

Title Length GC% TM Self Complementary 3' Complementary 3' Stability Repeat Seq Quality

rs1298467F 21 61.9 65.43 3 2 8.1 12 GGCAAGACACACTGGGAGACG OK

rs1298467R 24 45.8 64.49 6 0 8.2 12 TAGGTGAAGAGGAATGCATGGTTG OK

TITLE:rs1298467 NUM:1 SNP:0 Strand:1

rs1298467FG:TCTCTCGGGTCAATTCGTCCTT GCACTTGTGTTTGAGGAGGAACAACTAAGAAG 67.65

rs1298467FC:TGTTCGTGGGCCGGATTAGT GCACTTGTGTTTGAGGAGGAACAACTAAGAAC 67.60

rs1298467FP:AGATGTCACATAGTTTGAAATCCAGATCTG TTTTTTTTTTTTTTTTTT 64.73

>rs147361802
ttaaacaactaaaatttatttattttgtcacaattctggaggctggaattccaagattaaggtgttggcagggttagtttcctctgaaggctctctccttggcttgtagatggctctcttctctttgtgtctccacatggccttccctccacctgtctatgtccaaattttctccttataaggacaacagccatataaaattagagcccactctaatgaccttatttaaacttaaagacactatctacaaaaaaaaaagtcacattctgagttagtaggggttagaacttcaacatacaaattttgagggaacacaattcaccctataaaagcctagaaaagtatttattgtcacaactggaaggagggtgggtgttactggcacttagcgggtagagccagggaggctgctaaaccccccacaatctacaggacagcccccataacaaagagccatccatctcaaaatatcaatagtgtcaagactgggaaattctTTC[Y]TACTTCCAAAGCCAGTAGTTTGATATTTTTTATGCATAAAGAAGAGCAGAATATATTATTCTAGTGAATACTCATATCATGAATTATAGCACAGTCTTCGTTAGGACTCTGCAAATATCCATCTTCATATATCACACACACAAACAATATTTCTTCTCCAGATTTCAAAGCAGCTTCTTGGGAATATAGTGTAAATTGGGTTCTGACATTACTGCACCCCTAAGACAAATTTGGGAAAATTTGTGGGCATCaaagagccctgtaaactgtaaagttccaggcaaataagttactgttatAAGAAAAAAAAAGGAACCACCATATAAAATATTAGCACCTTTATCCTTAAAGAAAAAAATTAACTCCAGATGGATTGCAAAGAGGGAAAGACTGAGGCGAAGAAGTCCACTAAGATCCCACTGCTATTGCGTGTCTACTCCCAGAAAAGTGGGAAACTCAGGATTCCAACTGGAGTTGTGGAATCTGGGGCTGGGAGAGAG

OLIGO_Name Start Length Tm Gc com 3'com Rep seq

rs147361802F 354 22 66.73 59.1 2 1 0 CAACTGGAAGGAGGGTGGGTGT

rs147361802R 684 24 64.60 41.67 8 3 0 TCCCAAGAAGCTGCTTTGAAATCT

ProductSize:330,PAIR ANY COMPL: 4, PAIR 3' COMPL: 1,Score 3.80 Rank Left:1 Right:1

TITLE:rs147361802 NUM:1 SNP:0 Strand:1

rs147361802FC:TTCCGCGTTCGGACTGATAT TCAATAGTGTCAAGACTGGGAAATTCTTACC 67.84

rs147361802FT:TACGGTTATTCGGGCTCCTGT TCAATAGTGTCAAGACTGGGAAATTCTTGCT 66.08

rs147361802FP:TACTTCCAAAGCCAGTAGTTTGATATTTTTT TTTTTTTTTTTTTTTTTTTTT 63.43

>rs150850682
CTGCCTAGTGTAGATGCTGCGGCCCCTAGGGAGTTCCCATTTCTCCCCTGTAGGGCAGTTAGCTACCAGATTTCTGGGTATCTTGGTCCTTTGTGATTGATCCGACCGCTTGCTGTAACTATCTTGGCATCTTTCCTTGTGCCCTCCATGTGTCCTTCCTTAACTTTTGTGCCCTGGCTCCATTTTACAGATTCCCACCTCGGGTTGGGAGAGGACCACGGTGGCCAAAATTCTTAGCTTCTTCCTTTCCCTCATGCAGCCCATGGATAGCCAGCCCCAGAGGTAATGTCACAGGATGGGAAGTTTCCAGAGTGGGTGGGAGGTGGGTGGTTAGAGAAAGGCAGCAGGGGCCTCCCTGTGGATGTCAAGAATCttttttatttatttatttattttGTCCCACAGTTTAATTGGGGCCGCAGTTTAAGTAACTGTTCCTTTGATGCATAGGGGGGgtgtgtgtgtgtgtgtgtgtgtgtgAGAGTCGGGGATCGGTAGTC[D]CCCTATAAGCATTTATTTTTCTGTGGTTCTGACCTAACATTTTTTTATTTAGGATTATCACAAAATTATAAAACAGCCTATGGACATGGGTACTATTAAGAGGAGACTTGAAAACAATTATTATTGGGCTGCTTCAGAGTGTATGCAAGATTTTAATACCATGTTCACCAACTGTTACATTTACAACAAGGTGAGTTTTTCTGTGTGTTCATTTAGTAGGTGGGGAGAAACAGTAACTTCTATTATTGCTGGATATGTTGTCTACATAAAGTTTAAATCCTTTGCTACTGAAGGTGTTATCCAGGTAGGGTAGTCGGAGTCTTAAAAACCTGACTCTAGATGGTACTATTGAACACAGTGATGTGACTTCAGAGCTCTAGTTGAAGGTTATTTAGAACACTTCATACTTGGGGGTGGTGGTCCTGTTTCTTAGAAATCACCAGAGACCTGAGTAGACCAGGGATCTGTTTTCTTGTCAGCTCTCAAGTTTTTTCTTCTTT

OLIGO_Name Start Length Tm Gc com 3'com Rep seq

rs150850682F 288 25 66.36 48.0 7 3 0 TCACAGGATGGGAAGTTTCCAGAGT

rs17796690R 623 24 65.82 50.0 3 2 0 GCATACACTCTGAAGCAGCCCAAT

ProductSize:359,PAIR ANY COMPL: 6, PAIR 3' COMPL: 2,Score 1.24 Rank Left:1 Right:1

TITLE:rs150850682 NUM:1 SNP:0 Strand:1

rs150850682RA:TCTCTCGGGTCAATTCGTCCTT GTCAGAACCACAGAAAAATAAATGCTTATAGCGT 67.90

rs150850682RT:TGTTCGTGGGCCGGATTAGT GTCAGAACCACAGAAAAATAAATGCTTATAGCGA 68.65

rs150850682RP: GACTACCGATCCCCGACTCWCAC TTTTTTTTTTTTTT 65.19

>rs17796690
ctgtcatcccagcagtttgggaggccgaggcgagcagatcaggagttgaggagttcaggagttcaagaccagcctggccaacgtggcaaatacccatctctactaaaaaaaaaaaaaaaattacaaaaattagccgggcgtactggcgcacgcctgtgatcccagctactcgggaggctgagacatgaaaattgcttgaacctgggaggtggaggttgcagtgaactgagattgcaccactgcaccccagcctgggcaacagggcaaaactctgtctcaaaaaataaacagaaaaagcacattcaacttacaatattttccattgacgatgagtttatcaggaagtaacttaaggaacatctgtagtgagctcctcatcaccagaggtatgtaagcTTAGTACAGACACCATAGAGAGAAGCCAAACATCAGAAAAGGTTGGGCATCATGGCCTTTCCTACCACCAGCCTCGGATCTCACATGCCAGCTCTAACTGCTTT[R]GGACAATCATGAGCTGAGCCTTCCTGCAGCCTGGATGTCAGATTCCCAGCCAGACATGACATCAGTTCTGCCCTGACTTTGCACAAGGGCCTCTTGGACCTCCTACCTCCTACCTCCCACTTCCTTTCCACTGCTGCCTCCTGCCTTCCCAGGGGACACCAGGCCTGACCAAAGAGACTTGGAGCCTGGTTGTGAGATGGAGTTCCCCGGGGAACATTCAGCTGATGGCCTTCTGCCCGGCAGGAATGAGACAAAGGGGGCCAGGGACCCTGGGAGCCCTGTTCACACGCTCCAGAGAGTCTGCGTGGAAATTCCAGGAGGGTCACAGGAAAGCTTCAGATTTAGAACCACAGTGCTTTATTTTTTTCCCCTTTTCTCTTCTGTTCCATCCTTTCCCTCATGGTCGGTGGGGGTGTCATAGAAGTCTGGGAAAGGAAAGCAAGCATCCTCTTGGGCTGAGTCACAGAAAGAGCATGGAGAGACGGAGGGTATGGGGTAGA

OLIGO_Name Start Length Tm Gc com 3'com Rep seq

rs17796690F 254 22 65.34 54.55 4 2 0 GGCAACAGGGCAAAACTCTGTC

rs17796690R 596 22 65.42 50.00 6 2 0 AAGAGGCCCTTGTGCAAAGTCA

ProductSize:342,PAIR ANY COMPL: 6, PAIR 3' COMPL: 2,Score 1.24 Rank Left:1 Right:1

TITLE:rs17796690 NUM:1 SNP:0 Strand:2

rs17796690RG:TTCCGCGTTCGGACTGATAT GGAAGGCTCAGCTCATGATTGTACC 70.08

rs17796690RA:TACGGTTATTCGGGCTCCTGT GGAAGGCTCAGCTCATGATTGTGCT 67.99

rs17796690RP:AAAGCAGTTAGAGCTGGCATGTGA TTTTTTTTTTTTTTTT 64.53

>rs17814919
TTACACTTTGTAACTTATTAACCAATAACAGTGAATTATTATACCTTCAGTTTTTGAAAGTGCATCAGTGACTTATTGGCTCTTGAGAGGCTTGTCAGCATATAAATGAATGTTCTATGGCAGACGTTAAGGAAAGACAGATGACCAACTTTATGCTTTTGGAAGGCAAATAAATAAGCCATGTTATTGGGAGCTAAAATGAAGTCTTACATATTTCTCAAATTAATTCAGCATTTGGTCTTTTCACTGGTATCTGAGTTTTGAAAATCTCCCTTCTCTAATTGTTGACTCTGCTCCCTCTTCTGGAACCTTGGTTAAATTGAATGGGAAAACTGTTTACTTGAAATTGAATAATGTCTTTTCTGTGTTGGTCAATAAGTCCCAGATTGAAGTAAAGACAAACTTTAGCTAGAAAATTATTCCCATAGCATAATTTAAAAGTGCTAACTATGGCATGGGGAAATTTTTGTCTCTCATTTTTCCAATGCACATCAGTATGC[R]TAGGATTATTTTAGTTTAAAGAAAGGGAATGTTTAAAATAAACACATAGGTCATCAAATACAATTCAGGGGCTATCTTGTAATATTTTATTCTTTTGGAGAATTACATTTCTGTTTTTACTCCTTTGTTTTGGGTTTAAAATGCTTGAAAGCATTGCAAAAATAACAAGAACTCGTTAATTATATTCTGGGCACTTCAAAGAAGCTCAGAATACACTTTAAAAGCATTACATTCACATTCTCCATATCACTAAGGTAAGAAAGTGAAGATTGTTCTATTTCACGAAGGACATTTTCACTTTAAGAATAAGTTTATGTGTCTGTGCAGGCTCACATACTATTCAATTGCGAATCAGAGTAGAATCTGTGTCCTCTCAATTTGATTTTAAAATACAATCATATTTCTACATCTTTTACATTCAACGGGAAATTAACTAAAAAATTAATACGTTTTATTGGCTTCCTTTTATTTTTTTCTATGAGTTGTTCATTAATGTCTCC

OLIGO_Name Start Length Tm Gc com 3'com Rep seq

rs17814919F 450 23 66.17 43.48 6 1 0 TGGCATGGGGAAATTTTTGTCTC

rs17814919R 709 26 66.26 42.31 5 2 0 TGAGCTTCTTTGAAGTGCCCAGAATA

ProductSize:259,PAIR ANY COMPL: 4, PAIR 3' COMPL: 2,Score 5.43 Rank Left:1 Right:1

TITLE:rs17814919 NUM:1 SNP:0 Strand:1

rs17814919FG:TTCCGCGTTCGGACTGATAT CATTTTTCCAATGCACATCAGTATTCG 68.85

rs17814919FA:TACGGTTATTCGGGCTCCTGT CATTTTTCCAATGCACATCAGTATCCA 67.60

rs17814919FP:TAGGATTATTTTAGTTTAAAGAAAGGGAATGTTTAA TTTTTTTTTTTTTTTTTTTTTTT 63.41

>rs34060527
gagttcacgaccagcctgggcaacatagcaagaccccatctctacaaaaaattaacaaataaaaaattagctgggcatggtggcatgtgactgtggtcccagctgctcaggaggctgaggcaggaggatcgcttgagccaggagttcaaggttacagtgagctggctgtgccactgcactccagcctgggcaataggtgttgccctggaaagcaaggtcctggagagtgagaccctgtctctttaaaaaaaaaaaaaagaaaagaaaaTtattggtacttaactcataagtgactttgaggatcagatgagaaaagctgtgtaaaataaagcacttggcaagtgtggggtgcatcagaagcacccaatgacatgtgagctctcattatGATTACAGCGTGTCCCAATGTGCCTATTGCACAGTCAGCCGGACAGGTGATGGTTCTTACCTTTGTCACTCTTTTCCCAGTTTCTGCCCTCACCGAGGGGCCAAGTGACAGA[S]ACTCACAGGGATCCTGGTGTCTATGCCCCACCTGCCCGCAGGGCCTGAGCTGCTCGAGGCAAGGCTTGGGATTCTAGGCTGGGCAGGTCTTTTTTTTTTCCTCTCTTCCCTTTTCCCTGCAATGCTCTTGTTTCAGCCTCAAACTGCGTTCCCAGAGCTTGCTCAAAACTCCCCAGAGCGACCCTCCCTGGGGAGGAAGTGGGGAGCGTGTGAGGTTGGTGAACTCAGCTGGGGTGTCAAGGGGGTGGGAGGGCACTCTGGGAACAGAGGCCCAGGGAAGCTGTGCGACAAAGGGGACATGGAAAGGGCCACGCAAGGGCTCCCACAGCTTCCCCAGATGCCTCTTCAGACCTTTGCTCTCTCCCTGCTTCTGCTGTTCCTCCCATGGTTGGGCTTTGCTATGGCCCCAGCCCCATGCTaataatagaaatggcagccatttatcataggtttatgatgtgcctggaaatatgctcgcacttgacatctgcctcatttga

OLIGO_Name Start Length Tm Gc com 3'com Rep seq

rs34060527F 411 22 65.68 54.55 5 3 0 CTATTGCACAGTCAGCCGGACA

rs34060527R 533 22 66.48 59.09 4 2 0 GGTGGGGCATAGACACCAGGAT

ProductSize:122,PAIR ANY COMPL: 4, PAIR 3' COMPL: 1,Score 0.80 Rank Left:1 Right:1

TITLE:rs34060527 NUM:1 SNP:0 Strand:1

rs34060527FG:TCTCTCGGGTCAATTCGTCCTT CCGAGGGGCCAAGTGACACAG 68.62

rs34060527FC:TGTTCGTGGGCCGGATTAGT CCGAGGGGCCAAGTGACACAC 68.56

rs34060527FP:ACTCACAGGGATCCTGGTGTCTATG TTTTTTTTTTTTT 64.79

>rs3862218
aagtaaaaaccacctggccatattgtcaccacatttaggtggggtatactttttttacgacagccttcacgcctggcctctgtgtatgcatttgagtttattacccatgGTCTAGGAAGATGTAATAAACCAAGCCAGGGAAAGGTTTGGCTATCTCTCTTTAGTCACGCCTATCAGGTTTAGTCTCCCTTTGGGGTGGGGGCTCCCTTGTGGGGATTCCATATGTACATAACTGATAGGTGACTTCCAACACATGTTGGTGAAAATAGATACAGCTAACAAGTATTtatactagggtctatagtaagtgcttaaaatgagccaatttacttaatctttaaaaacctttgaggcgtaggtactgtcattctgaggcacaaaggagttgttacttgtccccataagcataggtgataagtggcaggattggaactgttttttgactctggagttagtgttcaccaactgctcttatgctgtCTCTGAAATAGACCCTAGCC[R]TTTTCAATTTGGTTGTTGTCCACATAGTTACAGATAATTTCTTAGGGTGATTATTTTTAGCCGTTGAAAGACTCTTAAATGATCCAAGTATGTCACTGGGGCTCTTGTCTGCTGACTTGCTAGGTCCTGGATTTGAAAAGTTTTCTGCCTTTCACTATCTACTTTGTGAAGTTTGGGATTGTTAAAAGTTCTAGTTTAGCATGTAGGTCAGCTATACAAATAAAAGGACTTGCTTAATTCCTGATACTGGGAGGTGAAGAATTAAAGGAGAGAATGTTGCACCTTATCTCCCTTAATCTTTTTtttttgtatggcctgcaaggtaagactgaatattgcctttttaaagggtGAtttttcttgagatggagtttcactcttgtcacccaggctggagtgcaatggcacgatcttggctcactgcaactccgcctcccaggttcaagcgattctcctgcctcagcctcccgagtagctgggattacaggcgcccgccacca

OLIGO_Name Start Length Tm Gc com 3'com Rep seq

rs3862218F 418 24 66.23 41.67 5 0 0 TGGCAGGATTGGAACTGTTTTTTG

rs3862218R 603 23 65.44 52.17 4 2 0 GCCCCAGTGACATACTTGGATCA

ProductSize:185,PAIR ANY COMPL: 4, PAIR 3' COMPL: 2,Score 3.79 Rank Left:1 Right:1

TITLE:rs3862218 NUM:1 SNP:0 Strand:1

rs3862218FG:TTCCGCGTTCGGACTGATAT TGCTGTCTCTGAAATAGACCCTAGACG 68.48

rs3862218FA:TACGGTTATTCGGGCTCCTGT TGCTGTCTCTGAAATAGACCCTAGACA 67.23

rs3862218FP:TTTTCAATTTGGTTGTTGTCCACA TTTTTTTTTTTTTTTTTTTT 63.46

>rs4132612
ATTGCTTTTATCCTTTCCTTTTTTTTTTTTTTTTTAATCTCATCCACCCTCATGACTTTTTCTGACTGTGAAAGAAAAATTCAGGTTGATAGCTTCTTGTGCTCTTAGAAGCACCAGTATCAGCCGTTGATTTGCTACAGTGGCAGGCAAGAGATGATGGATTTCAGGGGACTGAGAGAAGGTCGGGAATTCAACCTTCCTTCAGAGCCAAAAATACATTCAGGAAAACCCAAGATTTCAACACAACAAAAATGTGTTTCTTTTTTTTTTTTATATTAACTGAAAAAATAGTTCTACTTTAAGAACAAAAACTGATTTAAAGAATGGGCTTGTGTCTACCTGAAAATACATCCAAAATAATCTAATTTTCATGAGGCACCTCAGTACTCATCTTTGATAAGAAGCAGTTGGTTCAACATAATTGCAGGAAGGATCTTTCAGTCAGTGATGGCGATGAGGCTAATATCATTCCCTGAGAATAGTAGGTATCAATGATGTGA[M]TCTGTGTAGGAGAGGGGATTCAAAGTTAAATAGGGGGTCAAAGTACCACTTGGACTTGTGTTTCAAGCAGGAAGCCTCTGGAATTAGGTCCAGTGATCCACAGGGGAGAAGAGAGCTAGGTGAGCAGTTCTTGGAGCAGACAGTCTTTTAATCCTATTGGATGAGCCTGTATTATTGTCTCTGCAGATGTTCTGTACCCTTCTGCAAAAGAGGCAAGCTGGCTTTTCCCATAACTGATGAAATCAAGAGAGCAGGTCTGGAAAAAACAAGGGAAATCTCCTTATATCTTACTACGTGTAAAAAGATGATTCTGGAATTCCCGAGTGCCTACTGAATTAATAAACCAGGAAGCTGATTTATTTTACCACTCTAAATCCAACATGGAAGAAACCGTCAGACATCTACTACATGTCATCCAGCAGTACAGATTGGAGAAGTAAACTCTGTCAGCCCACCTTTATCAAACAGTGTAAATAATCACCAAACCAAGGGAAGCAGAA

OLIGO_Name Start Length Tm Gc com 3'com Rep seq

rs4132612F 441 22 65.19 50.00 5 3 0 TCAGTGATGGCGATGAGGCTAA

rs4132612R 610 22 65.91 59.09 6 2 0 TCTCCCCTGTGGATCACTGGAC

ProductSize:169,PAIR ANY COMPL: 7, PAIR 3' COMPL: 0,Score 0.90 Rank Left:1 Right:1

TITLE:rs4132612 NUM:1 SNP:0 Strand:1

rs4132612FC:TTCCGCGTTCGGACTGATAT TCCCTGAGAATAGTAGGTATCAATGATGTCAC 66.18

rs4132612FA:TACGGTTATTCGGGCTCCTGT TCCCTGAGAATAGTAGGTATCAATGATGTCAA 66.47

rs4132612FP:TCTGTGTAGGAGAGGGGATTCAAA TTTTTTTTTTTTTTTTTTTTT 63.30

>rs4705341
CATTCTGTTGTGTCCTGTCTCCCAGCAGAGACTACAAACTCCCCAAAACCACTTACCCACCAGCTGCACGTGAGAAGCCAAAGGTAGTTTATGTGAAAGGGCTTTGGAAATAATCACGCACCAAGTGAAGGCAGAGGACACACCTTGTCAGCTTAGTTCTCAGCAGCAAATCATCTCTTTTCCAGGATAACCCTCCCTGATTCTTATTGAAATCTCTTTGCTGACCACACTAAGCTCTTCTCTCTCAGGGGCAGTGGGAGCCGTGGAGAGTGGAATAGACCAGCTGTCTGTGACCTGCGAGGGAGTCCAATGTCGGAATCACTCCCCAGCCAAATGCACGGTTTTAAAAAATCTATTTATTTATTTATGTAGAGACCAGGCTATGAGActggctaatttttcgtatttttggatagagacaggatttcatcgtgttgcccaggctggtcttgaactcctgggctcaagcgatccacctgcctcggcttcccaaagtgctc[R]ggattacaggcgtgagccactgtgcccagccACCAAATGCAGTTGAAAAGAGTTTCTGCAAGATAATTCCACAGAAGAGGAAAGCAGTTATCTGGCTGGGAATACCTTAGACAGAGGCTGTCCTCTACACAGGCTGTCAGAACTGACCTACTGACCTGCCTGCTGGCATGATAGACCAGAGGGAAACACTCTTTCCACTCTTCCCAGACTGAGTGTAAGAGACAGACttttttttttttttttgagacggagtctcactctgtcacccaggctggagtgcaatggcaagatctcggctcactgcaacctccacctccccgggttcaaaccattctcctgcctcagcctcccaggtagctgggattacaggctcccgccaccacgcctggctaatttttgtatttttagtagagacagggtttcaccatgttggccgggctggtctcgaactcccaacctcaggtgatccgcctaccttggcctcccaaagggttgggatt

OLIGO_Name Start Length Tm Gc com 3'com Rep seq

rs4705341F 437 22 66.13 59.09 7 2 0 CCCAGGCTGGTCTTGAACTCCT

rs4705341R 668 22 67.19 63.64 6 0 0 GCCAGCAGGCAGGTCAGTAGGT

ProductSize:231,PAIR ANY COMPL: 5, PAIR 3' COMPL: 3,Score 1.32 Rank Left:1 Right:1

TITLE:rs4705341 NUM:1 SNP:0 Strand:1

rs4705341FG:TCTCTCGGGTCAATTCGTCCTT TCGGCTTCCCAAAGTGCACG 68.00

rs4705341FA:TGTTCGTGGGCCGGATTAGT TCGGCTTCCCAAAGTGCACA 66.42

rs4705341FP:GRATTACAGGCGTGAGCCACT TTTTTTTTT 63.72

>rs4760939
TCATTATCTTTTCTCAAGATAAATTTTAATGGAGGCCTGGTCTGAGAATACAATGCCATAAGTTCTGAATCTTCAGGTTCGTGCAACAAGTCATTTCTTAAGTGACCTTCAGCTGACTACATAAAATCTGAATTGGTCTTGATTAGAATCTGTCCAGCTGTCAGTCTTTTGGCCTCTGAGATACAGAGGGCATATACCCATGATGCTGTGGGGTTATGTAAGAGACAGTAAAGAAATAGGGGGGAAAAAAACAGAATAAAATGTAATTACACAGAGTTTCGTTTAAAACAAAAATTGATTGAGTTCTGCTTAAAGTGGCTTGTTGTTTCCTGTGCCAGGAACTACTGTGCAGGCCCAGCAGGAAGTGTGCTACAGAGCAGGATCACCTTACACCCTCGTTTTATGGTTCATTTCACTTATTTAATGCAATCTGAGAACCTGCAAAAGGAACATGTTAGAAGGAGGGAATAGGCTTTTTGTCTTGTTTTGTTGTTGTTGTT[K]TTTTAAATTTCTTTGAATCAAAACATTTGTTTTTACAAGGTGCTTAAAGTCCCAGAGAAACAGGACTTCTAGAGTTGCACAGTGAAGACATTTCTCTTTGTCACACAATTTAAAGCAAGACACAAAGTTGAGTTATGTCTAGAGTTGAGTTATGTCTGTTTCCTTTGCTGTCCTACTCACATAAATTTAAGCATAAAGAATTACAACCATGCCCATGAATGGTCTAAATCATTTTGAATTCCATGTAAGTTCCTCCTAGGGTCTCTAATTTGATAGAGAGCCCATGATGTTGTGGGGAATGTTGGAGAAAAGGAATTAATATTTAAGATGTAGAATAGCAATTCATTTGTCAAGCAGGGGAATGAGTTCATTTTACTGTATTGTTTGGAAATGTGTGAATTGAACAGCTGGATTTCCTTGTGGTGGCATTTGCTGCTCCTCACACGCTGTCTGTTTTCCCCCCCAGAGACCTCAGTATGAACAACATCAGTCAGCTGCTC

OLIGO_Name Start Length Tm Gc com 3'com Rep seq

rs4760939F 377 25 65.53 48.00 4 0 0 CAGGATCACCTTACACCCTCGTTTT

rs4760939R 722 24 64.36 41.67 6 2 0 CATTCATGGGCATGGTTGTAATTC

ProductSize:345,PAIR ANY COMPL: 6, PAIR 3' COMPL: 1,Score 7.11 Rank Left:1 Right:1

TITLE:rs4760939 NUM:1 SNP:0 Strand:1

rs4760939FG:TCTCTCGGGTCAATTCGTCCTT GCTTTTTGTCTTGTTTTGTTGTTGTTCTTG 67.20

rs4760939FT:TGTTCGTGGGCCGGATTAGT GCTTTTTGTCTTGTTTTGTTGTTGTTCTTT 66.05

rs4760939FP:TTTTAAATTTCTTTGAATCAAAACATTTGTTT TTTTTTTTTTTTTTTTTTTTT 63.11

>rs4854469
ATGAATAAATAACTGATGAAGGATATGTTTTCTGAGCAGGTTTCTGTGACTTATTGTTAATGCCAGTATCTAACACTTGAAAATAGTTTAGTGCTTTTAGTTAGCAGATTTTCTTTCTTACAGAAAAAGTATGATTTTTTGCCTACCCTATGTACATAATTAAATGTGTATAGTTGCTCTGGATTTCTGCTATGTTGTGATTGCTTTGTGATTGttctttttattattttatattttttaattatttatttatttatttttttgagacaggatctcgttttgtcacccaggctggagtgcagtggtgcagtctcagctcactgcagccttgacttcctgggctccagcaatcctcccacctcagcctcccaagtaggtgggaccacaggcccgcaccaccacacccagctaatttttgtatttttggtagagatgggattctgccatgttgcccaggccagtctccaactcctgggctcaagagatcctcccgcctcaga[M]tcccaaagtgctgggattacaggcatgagccactgtgcctgaccAATTGTTCTTTTTCTGCATGATGTTCTGTGTTAGAAGCCCTATTCATTCATTTGTTCACTCAACAAATGAGTTTGAGTGTCTGCATGCCGGGCTTTGCTTTGGACTTTGGTTACTCTTCTTTGCACTTTCTGAGCCTGTAGTGGGATAATAGCATTACTCTTCCAATTTCTACCTGAAAAGGTCAAGCAAATGCATATGTTTCCTGTCATATGTTTTTCTTCAAAATGGTTTTGTTTTTTTAACCAATTGCTTGATTATTTAAAGAGAAAAGTGAAAGTGAGGAATTCTTAAAGAATTGCAAGGTTAAGTCTAAGAATTCAAGTATATATGCCCCATATATATATAGATCCCTCTGCTCTTTAGCAAACTAGTCCTGATGAATATGAAAATTCATTTGCTGCTTACATGTTTATAGTCAAAAATTGTGGTAGAAGAGCATGATCCTGCATGGATGT

OLIGO_Name Start Length Tm Gc com 3'com Rep seq

rs4854469F 178 27 65.59 40.7 3 0 0 CTGGATTTCTGCTATGTTGTGATTGCT

rs4854469R 613 22 65.38 54.5 6 0 0 CGGCATGCAGACACTCAAACTC

ProductSize:459,PAIR ANY COMPL: 4, PAIR 3' COMPL: 1,Score 0.51 Rank Left:1 Right:1

TITLE:rs4854469 NUM:1 SNP:0 Strand:1

rs4854469FC:TTCCGCGTTCGGACTGATAT CAAGAGATCCTCCCGCCTCACAC 67.76

rs4854469FA:TACGGTTATTCGGGCTCCTGT CAAGAGATCCTCCCGCCTCACAA 68.10

rs4854469FP:TCCCAAAGTGCTGGGATTACAG TTTTTTTTTTT 63.45

>rs4971066
ttttgtttttttgttttttttctgagacagagtctcgctctgtcgcccagactggagtgcagtggcacgatctcgacttgctgcaacctccacctcccgggttcaattgattcttctgcctcagcctcccgagtagctgggactacaggcatgtgccaccatgcccggctaatttttgtatttttagtagagacggggtttcaccatattggccaggctggtctcgaactcctgacctcgtgatccgcccacctcggcctctcaaagtgctgggattacatgtgtgagccaccgcgcctggccTAGGAGTAGTATTAAGTGGCCCAGGCAAGAGGAACATATTCAGACTCGGAGGAATGAAGGATGAAATGTGGGGAGGGGCAGTGTCTATGCTGAGGGTTATTTCCAAAGAATGAGAGGCTGGGCTGAACATTGGGCAGGAAGTTAGCAAAAACTAAGGAGGGTAGGAATCAAGGTTAGAGGGAAGAGAAGAATGAAAT[K]GAGTAGGGAGCTGAGAAAGCAGGGCGAGGGGCATTTGGACTTACATTTTCTTCCAGCAAAGGTTTCTTATTTAACCCCTGTGGGCTTATCTTGCAGCCAAACCCATCCACCAGCATGAAGACCGCTGCTTGAGGTTGAAGGTGACTGTCAGTGGCAAAATCAGTGAGTGTCAGAGCCCTGTGGGCCTCCTTCCTCCATCTCTATGCTGGGTGCGGTCTAGTGATCTAGGATGGTATAGAAGTCTTGCAGCCCAGCCCACTCATACTTACAGCCCTCTGCCTCTTTGATACAGTACCTGATCTACTACCACTCTTGTCTTTCAGCTCACAGTCCTCAGGCCCATGACAATCCACAGGAGAAGAGACTTGCAGCAGGTGGGTAGCTGGAGCAAACTGGGCCTGGGGCACAGGAAGGGGTCTGCTTGAAGAGGTTGGGTACAGGAGGTGGCTCCTTTACCAGGCTGAGGCTGAGGGAATCCTGTTGGGCTGAGAGCCAGTACA

OLIGO_Name Start Length Tm Gc com 3'com Rep seq

rs4971066F 313 22 65.68 54.55 5 0 0 ATTAAGTGGCCCAGGCAAGAGG

rs4971066R 606 22 65.57 50.00 4 2 0 TGGGTTTGGCTGCAAGATAAGC

ProductSize:293,PAIR ANY COMPL: 5, PAIR 3' COMPL: 1,Score 0.75 Rank Left:1 Right:1

TITLE:rs4971066 NUM:1 SNP:0 Strand:2

rs4971066RG:TTCCGCGTTCGGACTGATAT CCCTGCTTTCTCAGCTCCCTACACC 68.50

rs4971066RT:TACGGTTATTCGGGCTCCTGT CCCTGCTTTCTCAGCTCCCTACACA 68.07

rs4971066RP:ATTTCATTCTTCTCTTCCCTCTAACCTTG TTTTTTTTTTTTTTTTTTTT 64.25

>rs530605210
TGGTCCTGAATGACTACTACATAGTAATTTGAAGCCCTCTTTGCCTTAGCTGTATGAACATTTCAGAATATAATTAGTTGTATATTACAAAGGAAAGCAAATTTGGGGAGATTTATGTTAATTATAATAAACTTAATTTAGAGCTTGAGCTAATACCTAACTACATTTAGAGTAAGTTCTGTGAAGAACTAAAAGGACAATCTTTTATCCACAAATTACCAGAATGACTAAGCTGTATATCTAAGGTCACATTACACAATGCAAGACTGAAATTTAATCGTGATTGCTAGAGCATTACCCTAATTGCCCAAATAAACAGAATTAAAATCACTTAGATGGGTTACAAAGGATTTCAGTGCATCAAAAGCTATGGCTACCCAGGgaatttagaatgggaagaaccttggaatttacctaatccaacgtctttatttcatagatgatgaaattttatattcataaggggttaatgacattctgaaaatcacgcagctagcaaa[R]acagaattggactccagatctcctgaaaaccccagcccagtactacttcttggtcaCCTTTATATTTCAATGTCAAATACTCGAATGAACTCAAAAACTCCCAGTTACTTTTCCAGATGTTGCCAAATAGCCCGTTGTTTACACATGTTGTGTATGTCCATATCTAGATATATACCTGAATTTTAACAATATCATCTTATTTTGATCCACTGTAAATACAGACACAGTGTCTCTGTATTACCTAAAAATCTGCAGAGCAAAAAGGAACACTCAGATTTAGTTTGAAGAATAATTAAGCATAATTAATTCCCCAAATGTCTGTTCATAGTTGTCATTTACTTCTtttttctttttttggtaaaaatgggagtatggctatgttgcccaggctggtctcgaactcctggcctcaagtaattatccacctcagccttccaaagtgctgggattataggcagtagccaccaggcccagccTCTGTTCATAGTAGTTAAATATGT

OLIGO_Name Start Length Tm Gc com 3'com Rep seq

rs530605210F 376 24 65.36 45.83 5 0 0 CCCAGGGAATTTAGAATGGGAAGA

rs530605210R 636 22 65.37 50.00 5 2 0 ACGGGCTATTTGGCAACATCTG

ProductSize:260,PAIR ANY COMPL: 3, PAIR 3' COMPL: 2,Score 2.73 Rank Left:1 Right:1

TITLE:rs530605210 NUM:1 SNP:0 Strand:2

rs530605210RG:TCTCTCGGGTCAATTCGTCCTT TCAGGAGATCTGGAGTCCAATTCTCTC 67.03

rs530605210RA:TGTTCGTGGGCCGGATTAGT TCAGGAGATCTGGAGTCCAATTCTCTT 66.47

rs530605210RP:TTTGCTAGCTGCGTGATTTTCA TTTTTTTTTTTTTTTT 63.09

>rs533309272
TCAACAATTAATGAATTAATCATTGTTTCTGGGGATGGAGACCATGAAGGTCTTCAGGTGATCTTATAAATATTAAACAGAGAGAACTACTTCAAAGTAtcccatccttgaatatgcattagaatcgcctagcacactttttaaagctactaatgaccagcggttgtccccagagattccgatttaatgggtctcactgggaacccagacatcttttatttcaaagctgtacaggtgggctgagaaccactgGAATATATGAAAGCACCCCAACAGACAAAGGCAGTCAGGGACTCATCAGTTGGTTGAACATAGGGACTAAAGGGGAGGTCTAAAGATGAGAGATGACTCTGAATAGGGTTATGAAATCGGGTGTCAGGAGCTGGTGAAGGACAAAAACAGGGAATTCAAgggaagaactgattttggaggcaagaggatgcccagttctggatatactaagtgcaaggaggcagcaggacatctaaatgggaatgtca[R]taacgttgttggaatgtaagtgtgaagcatgatgggtctgttctagagataatgattggagagtggtttacaaagaggtgatggttgaaggtgctgggtggatgagaaccctgagagagAAACTTGAGAGAGGCTCTgtgtttcccagtgtagtttttcaatctacaataaaatcactggagctgcttattaaaaatgcagatttccaagtccttctcccagaatgtctggttccctgggtcaggattggggccttggggatctgaatttttatggcttctaaagtgatggcaatgcacactaaaatttgagtaccactAAGCAGAGCAGAAAAAAAGAAAAGGTGTGAGGACAGAAACTTGAGGAACATCTATGGTTGAGCAATGAAGACTAAAAGAGAGCCTAGCAAAAGGAAGAAGAGATGACCAGTCATAGTGGTAAGAGTAGTAAGATTGTGCTGAATCTGAAAGGCAGTGAGAGGAGTTTAAATAAAAAGCAAT

OLIGO_Name Start Length Tm Gc com 3'com Rep seq

rs533309272F 265 22 65.69 54.55 2 1 0 CACCCCAACAGACAAAGGCAGT

rs533309272R 598 22 65.70 54.55 3 0 0 CCAGCACCTTCAACCATCACCT

ProductSize:333,PAIR ANY COMPL: 4, PAIR 3' COMPL: 1,Score 0.61 Rank Left:1 Right:1

TITLE:rs533309272 NUM:1 SNP:0 Strand:1

rs533309272FG:TCTCTCGGGTCAATTCGTCCTT AGCAGGACATCTAAATGGGAATGTGAG 66.47

rs533309272FA:TGTTCGTGGGCCGGATTAGT AGCAGGACATCTAAATGGGAATGTGAA 66.73

rs533309272FP:TAACGTTGTTGGAATGTAAGTGTGAAGC TTTTTTTTTTTTTTTTTTT 65.26

>rs66814253
cccaggagctgaatcttgaagcacaactaggaattagccacccaaataagatggccgactgaggggacaatacgtacagcaacacagagtcagaaaggacctgggatgaacaggtagctccaagcaggttagtacttgagcattcaggagaagctacagagcatttggagaagagcttgtaaagacagaaggagtcCCAGTTGCAGGGATCCCATATGTTAAGTCACATGGGTGGACATTATGAGGAGGCATGATGAGGAGTCAAGGAAAAGATGTACATAAGTGAGGGAAGGCCATGTTCAGACTTTGCACTATAGCTGGGTCCCTGTCAGTGTGAGGAGAGTTCAAGAGGAACAAGATCCTGCCAGAAATTGTGTTCACTCTTCTTTTTACTTTGCTGACCTAAAGACTAATATTCAATGAATGGACTCAAATTGTCAAATATTTTTAAAGTATTTTGGTTTTtgtcttaggttgtttgtgctatgataacggaatac[Y]gcaaactgggtaatttataaagaagagaagtttatttctcacagttctggaggctgggaagtccaagatcaaggtgctgacatctggtgagggctgctttctgcttccaagatggcatcttgagagccaaatcctctggaggataggaatgctgtgtcttcctatggcagaaggtggaaaggcaaaaaaggaataaacttcttccaacaaggctttttatgatggaattaatctatttcatATGATTGTACTCCAAACATTTTTAAGGCGATAATACAAAATATTTATAAAGCAAACTTTTGAGGAAATTCATTGAGATCACCATTTCTTATTTTTCTGCTAATTTAATTAAAACATCTCCCAAAGGCTCTacccaacacctcccaaaggtccccacctcccaacactattgcattggggattaagtttcaacatgaatttttggagaggacaaaaacattcaaaccataacaGCATTAGAATAATTTttctcagcaatg

OLIGO_Name Start Length Tm Gc com 3'com Rep seq

rs66814253F 283 22 65.82 54.55 4 1 0 TGAGGGAAGGCCATGTTCAGAC

rs66814253R 609 22 66.78 59.09 3 0 0 GGAAGCAGAAAGCAGCCCTCAC

ProductSize:326,PAIR ANY COMPL: 6, PAIR 3' COMPL: 2,Score 0.96 Rank Left:1 Right:1

TITLE:rs66814253 NUM:1 SNP:0 Strand:1

rs66814253FC:TCTCTCGGGTCAATTCGTCCTT TCTTAGGTTGTTTGTGCTATGATAACGGAATTCC 68.51

rs66814253FT:TGTTCGTGGGCCGGATTAGT TCTTAGGTTGTTTGTGCTATGATAACGGAATCCT 66.95

rs66814253FP:GCAAACTGGGTAATTTATAAAGAAGAGAAGTT TTTTTTTTTTTTTTTTTTTT 63.33

>rs72865868
GTGGGAAGATTTGTCCTTCAAACCTTTTACAGCCAATGGGAGCGTGGAGGGGGGGCGAGCGGGAGAGGGCCATGGGGGGGGAGGGGAATGGCCAGCCTCATGCCTCCGTACCCATTGGAGGGCAAAGGGGTTAGGGGGCGGTGTGGCCCCCCCTATTCCATTCGTCCCCTGGGGGTACAGCAGCCGGGAGCCAGGTGAGAAGGGATCCATCGGCGGCCGAGGGAGGGGTGACCTGGCGGTGGGCTGAGGAGTGGTGGCTGTGGCCCCTACCCGTGGATGTGAATGCTTTAGGAGTTGGCCACCCATGTTGTGAACTGAGGTTGTTCCCAGGCGCCAACTTCCTTTCTCCCCAGAGCCTCTGGAGGGAGCATTGCTGTGCGCCCTTTGTGTCCGCGGTAGGGGAGCTCCAGTCGTCACACCGCAGGCTGGAGGTTACGCTTCGAGTCGCTTACCGAATTTGTGTGCATTCACGTGGACACGGCCTGTGGGGCCTTTTGCCC[Y]TGTAGGGTCTTTACTGAGCACGTGTCTACTCCAGGCTGGGGTGCTTACAAGCTGAAAGCTTGAGGTCTGCTTAGGAACAGAAACCAGGCCCAAGGTGGGTGCTGGCAGTAGGGGGTCTAGACAGCATGGTCTGAGATGCGAGGGAGGCTCGGGACCTGGAATGATTTCACAGCTCCCAAGGTTTCGGGTTTCTCCAGGGTGGCCTCTTCCATCGCCTCCCTCATCCCCTCCCCCAGTCCTGAACAGTTCTCTCCTTGTGTACTGCGGGGGAGGGAACGGAAAGGAGGAAAGAGTTACTTTCCCAAATTACTGAGTAGCAGTAGCCTCCCTGGTGACTCATGTGGGGGAAGGGAGGATAGAGGATCGGGAGGCAGTGATTTTCCGGAATGCAGGGAATAAACGAGAGCAATGTCTGGCTGCCCTTTTCCTAAGGCCTAGTATTTTCTCAGCCTCCTAAGTTTTTATTCCATGGCCGGCCCCCTGATGGGCCTCTGTCCTGG

OLIGO_Name Start Length Tm Gc com 3'com Rep seq

rs72865868F 456 22 65.74 45.45 6 3 0 TTTGTGTGCATTCACGTGGACA

rs72865868R 770 22 65.90 59.09 4 0 0 CCCCGCAGTACACAAGGAGAGA

ProductSize:314,PAIR ANY COMPL: 6, PAIR 3' COMPL: 0,Score 0.36 Rank Left:1 Right:1

TITLE:rs72865868 NUM:1 SNP:0 Strand:1

rs72865868FC:TCTCTCGGGTCAATTCGTCCTT CCTGTGGGGCCTTTTGCACC 71.90

rs72865868FT:TGTTCGTGGGCCGGATTAGT CCTGTGGGGCCTTTTGCACT 69.65

rs72865868FP:TGTAGGGTCTTTACTGAGCACGTGT TTTTTTTTTTT 63.38

>rs7308513
acatagtgagacccctgtctctctataaaaaattaaaaagttagctgggcatggtggcctgcgcctataatcccagctacacatgaggctgaggtgggaggatcacttgaactcaagaagaattcgagggtacagtgagccctgatcatgccactgcactctctgaggataggactcatgcatgcactagtgttttctaaCTGGCTTAATTGCACAGTTAAGTATTTCCTAAGTGAAAATTAGGAAAACTATTGACCTGACCTGGAGCATTCCCTCAGATGATCTGCAAAGTATTCTCCAGAACTCTTTTCAAACCTGAAATTacaagcttgagtaatgcagtgagatgctgtctctaaaaataattaagtaaataaataaaattggctgggcgtcatgtgcatgactgtagtcccacctatgctggaggctgaggtgggaggatcatttgagcccaggagtttgaggctgcagtgagctatgatcctgccactgcactc[Y]agcccgggcaatagagcaagaccgtgtctctgaaaaaaaggaaaagaaaTACCTGAAATAATAACAAAATCCATACTTTGCACTCTTCTATGAGCTAATAATATACTTAAAATATAAAAGTCAGGCATTGTCTTATTTGTGTTTAATTGAAAACAGCTTGCATGCCTATGCATTCCACTGATAACCCAGGGAGATAAAGTTAAGAGGCCACATGCTCTATCAccctgctattcagatttgttctgtgggccagcagcatcagcactgcctaagaaatgcagaacacttggtctcagctctgtcttacttaaaaaaatcttcattttaacaaaatcctaggtgattcgtaggcaaaatgcatttgagaaATTAAAATTGTTAGTGTTCTATAACACTAACAAAGTGGTCTACAAATCAAAAATGGTTGAAAACGACTGACGTAGATCATTTGCATTCTTTGCTCCACCCATATACACTTGGCTTCCTTGAATAAAGTTTAG

OLIGO_Name Start Length Tm Gc com 3'com Rep seq

rs7308513F 258 23 66.47 56.5 4 3 0 GACCTGGAGCATTCCCTCAGATG

rs7308513R 756 22 66.24 50.00 5 2 0 TGCTGGCCCACAGAACAAATCT

ProductSize:498,PAIR ANY COMPL: 5, PAIR 3' COMPL: 2,Score 0.46 Rank Left:1 Right:1

TITLE:rs7308513 NUM:1 SNP:0 Strand:2

rs7308513RC:TTCCGCGTTCGGACTGATAT TCTTGCTCTATTGCCCGGGATG 69.61

rs7308513RT:TACGGTTATTCGGGCTCCTGT TCTTGCTCTATTGCCCGGGGTA 67.11

rs7308513RP:GAGTGCAGTGGCAGGATCATAGC TTTTTTTTTTTTTT 65.48

>rs78278727
CCAGATTAGGCATGATTGCAGTAAGAGAAGAGACAGACCCTTTGGCCCCCCACCCCTGCTCAGGCTCAAAAATGCAGACCCTGCCGAAACAGTCCTTCTCACCCAGAAGCACCCCATAGGGTGGGCTGAGTAACCTTGGGGGCCTCGTCAGTCTTGGGCTGCCCCATGCCCTGCACAGCCCGCCTGAGGTTTGAGGAAGGGGCAGTTGGCTAGGCCCAGACTGGAGAAAGCCACCCCACCATGGCTCTTCTGCAAGAACCCCCGGCCAGCCACAAGCCTAAGCCCCCTCCTTAAAAGCTCCTCCTCTGACCTTAGCTGTGCATCAAGGGAGAAAAGAAAGCTCCAggccgggtgcggtggctcacacctgcaatcccagcactttgggagaccaaggctggcagatcattaggtcaggagttcgagaccagcctggccagcaaggtgaaaccccatctctactaaaattacaaaaaattagtcaggcatggtgacacgtg[Y]ctgtagtcccagctactctggaggctgaggcaggagaattgcttgaacccaggaggcgaaggttgcagtaaaccaagatcacgccactacactccagcctgggcgacagagcaagactctgtctcaaaaagaaaaaaaaaaagaaaGCTCCAAACTGCTCTGCTGCCACATACTCTACTCCTCCTGTCCCTCCAAGGAGGCAGGGATGGGGGTGGAGTGTCTAGAGGGAGGCTGCCTGCTGGCCTGGGGAGGGATCCACAGAGGCTATGACACCACCCCTGGCTGGGCTACTGGGCTCAGAGGCCCCCGGTCAGCCTGCCCAGAGCCTGCCAGCCACCAGGCATGTGACCCAACAACCCCGATGAGCTGAGCAAGGCAGGGTGAAGGAGAAAGAGGCTGGCTAGGACCAGGCCCTGGCTCTGCAGCCAGCACAACTGGGTAACTGTCCCACTCCTCTGGGGCCTCAGGCCATAATGTTCCCTGGTTTGGGACAGAGGGGA

OLIGO_Name Start Length Tm Gc com 3'com Rep seq

rs78278727F 295 25 65.62 56.0 4 2 0 AGCTCCTCCTCTGACCTTAGCTGTG

rs78278727R 676 24 66.14 58.3 6 2 0 CTCCTTGGAGGGACAGGAGGAGTA

ProductSize:405,PAIR ANY COMPL: 5, PAIR 3' COMPL: 1,Score 0.38 Rank Left:1 Right:1

TITLE:rs78278727 NUM:1 SNP:0 Strand:2

rs78278727RC:TTCCGCGTTCGGACTGATAT GCCTCCAGAGTAGCTGGGACTACTGG 68.40

rs78278727RT:TACGGTTATTCGGGCTCCTGT GCCTCCAGAGTAGCTGGGACTACTGA 67.19

rs78278727RP:CACGTGTCACCATGCCTGAC TTTTTTTTTTTTTTTT 63.73

>rs78280497
CGGGGCCGCCATTACAATCCACCTCCATCCGCTTGGAAATGGCCTTCGTCCCGGCCTATGACTGGTCCCAGCGGGCAGTACAGACCCCCTAGAAGCCCCTGGAGCTCCCCTTTTTCGGGCCCCGCCCAATCCTCGGAGTCTGTCCACCCCCTCTACTCCGCCCTCAAGAGGATTTCAAAGATGGAGGCGGCGGCTCCCTAAACCACTTTTCGTGTTCATCCGCCTCCATCCGAGATCGAAACGGGACCTCGTCGGCCCCGTAGGGGCCCGACAAGAAGAGGGAATCCCTGCAGACCAACAGCGGGCTATATTGACGACGGTGTCTGAGATCGGGGACCGTCTTTTGAAGAGTCAGTCCCTCCTTAGTTGCCCGCCTCAGCTGAGGCCGCCGCCATTTTCTTGCTGTCCGCCGTCTGCAGAGCGCGCCAAGCTGCCCGGAGCTCTCCGAGAGGCCCCAAAGAGACTGCTTTCGTGCCGGCCAGGCAGGGGGTTTGTCGCCT[S]GAGGCCCAAGAGGAACGGCCTCCCCCCAACTTAGCGGGTTATGCTGGACCGGGCGGTGAGGGGAACCGAGGCCACCCGGACTTTCCGCGGCTGAGGGCAGCGCCGGTTCCTTGCGGTCAAGATGCTGCAAAACGTGACTCCCCACAATAAGTACGTTTCCGCGAGCCGCGTGTGGGAAGGGGATGTTGCAGGGCGGCGGCACAGGGGTGTGGGGCGCCGTGTTGGGAGTACTGAGCGGCCCCGGCGCGCTGCTGTTGCGGCGCAGCTGTCGACTCGGTCGCGCGGAGGGAATTGAGCGACGGTTTTGGAACGGTGGTGGCGGCTCGGCTACTGCTCGTGGAGGGGAATACAGGTTGTCAATTTATACGCTATTAATGCCGCCGTGGCCCAGTCTTAACCGAGTCAGGCAGAGCTAGTTTGACGGTGGAGTGGAGTGAGGTTGAACAGCAGGTTTGGCGTTTGGTGGGTCTGGTATCTAGCGGCGGTCTGTTAGCCTTTTA

OLIGO_Name Start Length Tm Gc com 3'com Rep seq

rs78280497F 321 22 66.07 59.09 5 3 0 GTCTGAGATCGGGGACCGTCTT

rs78280497R 628 22 66.35 54.55 5 1 0 CAGCATCTTGACCGCAAGGAAC

ProductSize:307,PAIR ANY COMPL: 4, PAIR 3' COMPL: 1,Score 0.42 Rank Left:1 Right:1

TITLE:rs78280497 NUM:1 SNP:0 Strand:1

rs78280497FG:TTCCGCGTTCGGACTGATAT GGCAGGGGGTTTGTCGCATG 71.29

rs78280497FC:TACGGTTATTCGGGCTCCTGT GGCAGGGGGTTTGTCGCATC 70.39

rs78280497FP:GAGGCCCAAGAGGAACGGCC TTTTTTT 69.54

>rs7976133
aaaaaagctaatgcgtccggggcttaatacctaggtgatgggctgataggtgcagtagaccaccatggcacacgtttacctacgtaacaaacctgtacattctgcacatgtaccccagaattttaaaaaaataaaaTAATTTTTTTTAAAGTGCTTGGTCAGTGTTAGCCCTGAAAATCATTAGGTAGAAGGCAACACTATGCCTTTATGTAGAAGGTCTTTTGTTCCCTCTATTAAGAGGTAGACAATGAAGGAGTATCCCCTTTGCAGCCTGCAGAGATGAGTAAACCAATTATCAGCTTTGATAATATGTGTGTCCATTTTATTATATTCGCTATTTTTCAAAAATACATACCATATTAATAATCAGGTATATTTGTGCATAGGTGTGAGATTTTGGAGGTATTGGGTGGGGGGGTATCTTTCAAACCTTCAGAAAGTTACAAATTTGGATTTACAAGCAGCTTCATTTGTACTATTTATGCGGAGTAAATGGACTT[Y]ATTCCTTTATAAATCCCATTTATTCAATAAATGTAGGATAAAGTGATCAATTATAAGACTAATTTGAAGCCCCTTAGAAAAAGTATGATTTTTCCCATTGCATAGTGGTGATCTGGTTTTAAGAATTAGAATCAGGCATGAAAATGTGTTAGTTGATTAGCTGATAACTTTTAAAACATGAAACAAAATTGTTCTTATATATCTATGTTACGTTATCTACCAACTTTCTTTTCTTTCAAGCTTTCGCAAATTATGATGGCTTGCTTTAGATTAGTTATATTATACTATAAATATGTCCAGATGTTTTTGTTATTCTGTAATTTGTTGAATTTTGGCTGAAGATTTTCTTAGATTCCGTTCCCCCACCTCCTTCTTGTAGTGATTGATTTCAAAGCTCCTGCTATGTTTTGAATTGTTAGGTTTTTTTAAAGAGTTGATTAGAAAAGAAACAACAATGATTTGTCTGAGCTGTAGAGATGTAGCAGCTCTTAGAGACAGAG

OLIGO_Name Start Length Tm Gc com 3'com Rep seq

rs7976133F 241 27 65.54 44.4 6 2 0 TAGACAATGAAGGAGTATCCCCTTTGC

rs7976133R 612 25 65.09 40.00 4 0 0 TCACCACTATGCAATGGGAAAAATC

ProductSize:371,PAIR ANY COMPL: 4, PAIR 3' COMPL: 1,Score 3.91 Rank Left:1 Right:1

TITLE:rs7976133 NUM:1 SNP:0 Strand:1

rs7976133FC:TTCCGCGTTCGGACTGATAT CATTTGTACTATTTATGCGGAGTAAATGGACATC 66.75

rs7976133FT:TACGGTTATTCGGGCTCCTGT CATTTGTACTATTTATGCGGAGTAAATGGACGTT 66.33

rs7976133FP:ATTCCTTTATAAATCCCATTTATTCAATAAATGT TTTTTTTTTTTTTTTTTTTTT 63.01
